# Supplementary material for: Autologous neutralizing antibodies and polyfunctional T cells contribute to long-term HIV-1 post-intervention control
Source: Nat Immunol. 2026 Mar 3;27(4):812–26. doi: 10.1038/s41590-026-02448-z (PMC13043296; doi:10.1038/s41590-026-02448-z)
Supplement: Supplementary file 1 — Supplementary Notes, Supplementary Tables 1–11 and Supplementary Figs. 1–12. [file 41590_2026_2448_MOESM1_ESM.pdf]

nature immunology

Supplementary information

https://doi.org/10.1038/s41590-026-02448-z

Autologous neutralizing antibodies and polyfunctional T cells contribute to long-term HIV-1 post-intervention control

In the format provided by the authors and unedited

Supplementary Notes

Modified IPDA: detailed methodology

Primer/probe sets targeting the φ-region and the rev-response element (RRE) within *env* were used to identify proviruses that are likely to be genetically intact in a duplex ddPCR assay[1], under the assumption that proviruses that contain both regions are highly likely to be genetically intact in the full HIV-1 genome. In parallel, two regions within the human *RPP30* gene were also assayed to allow quantification of the input number of cells and an estimation of the level of DNA shearing within the sample, which can bias the quantification of intact HIV-1 genomes. Briefly, 10-50 x 10^6 PBMCs were subjected to negative selection to isolate CD4 T-cells (CD4 T-cell isolation kit, Miltenyi, 130-096-533), and genomic DNA was then extracted from the cells (DNeasy Blood and Tissue kit, 69504; or Qiamp DNA Mini Kit, 56304; Qiagen). Each ddPCR reaction contained genomic DNA, ddPCR Supermix for Probes (no dUTPs; Bio-Rad, 1863024), primers (final concentration 900 nM), probes (final concentration 250 nM), XhoI restriction enzyme (ThermoScientific, ER0691) and nuclease-free water. All primer and probe sets can be found listed in Supplementary Table 7. Droplets were prepared using a QX200 Droplet Generator (Bio-Rad, 1864002) and cycled at 95°C for 10 min; 45 cycles of (94°C for 30 s, 59°C for 1 min) and 98 °C for 10 min, using a ramp rate of 2°C to improve droplet separation. Droplets were analysed on a QX200 Droplet Reader (Bio-Rad, 1864003) using QuantaSoft software (Bio-Rad, version 1.7.4), where replicate wells were merged before analysis.

qVOA/Q^{2}VOA: detailed methodology

For ID107 and ID142, qVOA was performed to quantify the frequency of inducible provirus. Briefly, on day 0, approximately 500 x 10^6 PBMCs from each individual and timepoint were subjected to negative selection to isolate total CD4 T-cells (CD4 T-cell isolation kit, Miltenyi, 130-096-533). Then, CD4 T-cells were seeded at 50,000 cells/well in round-bottom 96-well plates, cultured with irradiated allogeneic PBMCs from HIV-negative healthy donors (10,000 cells/well) and stimulated with phytohemagglutinin (1 µg/mL) (Remel™ PHA Purified, Thermo Scientific™, R30852801) in a total culture volume of 200µL. Culture media contained RPMI with L-glutamine, 1% streptomycin and penicillin, 10% fetal calf serum, recombinant human IL-2 (100 U/mL) (Gibco, PHC0027), and conditioned media from a mix lymphocyte reaction culture[2,3]. On day 2, the PHA was washed away and 10,000 MOLT-4/CCR5 cells were added to each well. On days 5, 7 and 9, the culture media was replenished with fresh media. On day 9, the cultures were also supplemented with an additional 10,000 MOLT-4/CCR5 cells. On day 12, 50µL of the cell supernatant from each well was incubated with TZM-bl cells (10,000 cells/well, cultured in 200µL/well DMEM + 10% FBS). On day 15, TZM-bl culture wells were assayed for luciferase activity, indicative of replication-competent HIV based on activation of the firefly luciferase reporter gene[2], using the Britelite™ plus Reporter Gene Assay System, 100 mL (Perkin Elmer, 6066761). Estimated frequencies of cells with replication-competent HIV were calculated using limiting dilution analysis as previously described[4]. MOLT-4/CCR5 (Cat No 4984) and TZM-bl cells (Cat No. 8129) were obtained from the NIH AIDS Reagent Program.

For ID9254, the similar quantitative and qualitative viral outgrowth assay (Q^2VOA) was used to quantify the inducible proviral reservoir. Briefly, CD4 T-cells were isolated from PBMCs

and cultured in media containing 1 μg/mL PHA (PHA; Life Technologies) and IL-2 (100 U/mL), and cocultured with 1 × 10^6 irradiated PBMCs from a healthy donor in 24-well plates. After 24 h, the PHA was washed away and 100,000 MOLT-4/CCR5 cells were added to each well. Then, the MOLT- 4/CCR5 cells were split in half on day 7, as well as day 9, 11 and 13. On day 14, cultures were assayed for p24 by ELISA to identify positive wells. Estimated frequencies of cells with replication-competent HIV were calculated using the IUPM algorithm (http://silicianolab.johnshopkins.edu).

Near-full-length sequencing of HIV-1 RNA from qVOA cultures: detailed methodology

Near-full-length (~8.7 kb; HXB2 coordinates 817-9501) HIV-1 RNA genomes were sequenced using a previously described assay based on single-genome sequencing: plasma-derived RNA using long-range sequencing (PRLS) assay[5]. Briefly, qVOA culture supernatants were subjected to RNA extraction using the QIAamp Viral RNA Mini Kit (Qiagen), with RNA eluted in 35uL elution buffer. Then, 16uL RNA was used as input into two cDNA synthesis reactions per sample. cDNA synthesis was then performed as previously described[5]. RNA was DNase-treated using ezDNase (Invitrogen, 11766051), followed by inactivation at 55°C for 5 min in the presence of 10 mM DTT. Then, RNA was denatured in the presence of 14 μM HIV-1-specific anchored oligo(dT) primer (5'- TTTTTTTTTTTTTTTTTTTTTTTTGAAGCA)[6] and 2.8 mM dNTP (Invitrogen, 18427089) at 65°C for 5 min. The denatured RNA was then mixed with a buffer mix consisting of 12 μL 25 mM $MgCl_2$ and 6 μL 10× PCR buffer II (Invitrogen, N8080130) plus 0.2% Tween and 40 U AffinityScript Multiple Temperature Reverse Transcriptase (Agilent Technologies, 600105), and cycled at 42°C for 5 min, 55°C for 1.5 h, 70°C for 15 min.

cDNA was then diluted to limiting dilution, and subjected to two rounds of nested PCR as previously described to amplify near-full-length HIV-1 RNA genomes. For the first round of PCR, the following primers were used: RNA F1 (5'- TTTTGACTAGCGGAGGCT-3') and RNA R1 (5'- GCACTCAAGGCAAGCTTTATTGAGGCTTA-3') in a 40 μL reaction consisting of 2 μL diluted cDNA, 1 μM primers, 1× High Fidelity buffer (Invitrogen), 2 mM $MgSO_4$ (Invitrogen), 0.2 mM dNTPs (Promega), and 0.025 U/μL Platinum Taq High Fidelity (Invitrogen, 11304011). PCR cycling conditions for the first round were 94°C for 2 min, then 94°C for 30 s, 64°C for 30 s, and 68°C for 9 min for 3 cycles; 94°C for 30 s, 61°C for 30 s, and 68°C for 9 min for 3 cycles; 94°C for 30 s, 58°C for 30 s, and 68°C for 9 min for 3 cycles; 94°C for 30 s, 55°C for 30 s, and 68°C for 9 min for 21 cycles; and then 68°C for 10 min. For the second round of PCR, the following primers were used: RNA F2 (5'-GCGAGAGCGTCAGTATTAAGC-3') and GlobalR (5'-GCRGCTGCTTATATGCAGGATCT-3') in a 30 μL reaction consisting of 2 μL diluted cDNA, 1 μM primers, 1× High Fidelity buffer (Invitrogen), 2 mM $MgSO_4$ (Invitrogen), 0.2 mM dNTPs (Invitrogen, 18427089), and 0.025 U/μL Platinum Taq High Fidelity (Invitrogen, 11304011). PCR cycling conditions for the first round were 94°C for 2 min, then 94°C for 30 s, 64°C for 30 s, and 68°C for 9 min for 3 cycles; 94°C for 30 s, 61°C for 30 s, and 68°C for 9 min for 3 cycles; 94°C for 30 s, 58°C for 30 s, and 68°C for 9 min for 3 cycles; 94°C for 30 s, 55°C for 30 s, and 68°C for 9 min for 31 cycles; and then 68°C for 10 min. Between 3 and 10 PCR positives were chosen for sequencing per qVOA culture. PCR positives were sequenced using an Illumina Miniseq. Pooled libraries were subjected to 2 x 150bp paired-end sequencing using the MiniSeq High Output Kit (300 cycles) (Illumina, FC-420-1003). Following sequencing, raw

reads were demultiplexed, and de novo assembly was performed using an in-house pipeline as previously described (https://github.com/laulambr/virus_assembly)[5,7].

<u>Single-genome sequencing of proviral *env*: Detailed methodology</u>

Briefly, resting CD4 T-cells were isolated from cryopreserved PBMCs using the EasySep Human CD4 T Cell Enrichment Kit (STEMCELL Technologies, 19052), followed by negative selection for resting CD4 T-cells using microbeads targeting CD25, CD69 and HLA-DR (CD25 MicroBeads II, 130-092-983; CD69 MicroBead Kit II, 130-092-355; anti–HLA-DR MicroBeads, 130-046-101; Miltenyi Biotec). Genomic DNA was isolated using the QIAamp DNA Mini Kit (Qiagen, 51306). Proviral *env* DNA was then amplified using a two-step nested PCR protocol targeting HXB2 5983-8882 as described previously[8]. The outer PCR used the following primers: envB5out (5'-TAGAGCCCTGGAAGCATCCAGGAAG-3') and envB3out (5'-TTGCTACTTGTGATTGCTCCATGT-3'), in a 10 μL reaction consisting of 1× High Fidelity PCR Buffer, 2 mM $MgCl_2$, 0.2 mM dNTP, 0.2 μM primers and 0.4 U of Platinum Taq DNA Polymerase High Fidelity (Invitrogen, 11304011). Cycling conditions were 94 °C for 2 min, 44 x (94 °C for 30 s; 50 °C for 30 s; 72 °C for 2.5 min) and 72 °C for 3 min. Outer PCR products were diluted 1 in 2 with 10 mM Tris-HCl, then 2 μL was used as the template for the second round, inner PCR using the following primers: envB5in (5'-TTAGGCATCTCCTATGGCAGGAAGAAG-3') and EF_inner (5'-TTTGACCACTTGCCACCCAT-3'), in a 20 μL reaction consisting of the same components as the first round. Cycling conditions were 94 °C for 2 min, 41 x (94 °C for 30 s; 55 °C for 30 s; 72 °C for 2.5 min), and 72 °C for 3 min. PCR products were subjected to 1% agarose gel electrophoresis. PCR products from plates with less than 30% of wells positive

(limiting dilution) for the ~3 kb amplicon size was subjected to Sanger sequencing using previously described SGS primers[9] (Azenta Life Sciences).

Single-genome *env* sequencing of positive modified qVOA cultures and plasma-derived genomes: detailed methodology

Following cDNA synthesis, single-genome amplification (SGA) of HIV-1 *env* was performed at limiting dilution as previously described[10]. PCR reactions were conducted using Platinum Taq DNA Polymerase High Fidelity (Invitrogen). The outer PCR contained 1× High Fidelity PCR Buffer, 2 mM $MgCl_2$, 0.2 mM dNTPs, and 0.2 μM each of VIF1 (5'-GGGTTTATTACAGGGACAGCAGAG-3') and OFM19, and was cycled as follows: 94 °C for 2 min; 35 cycles of 94 °C for 15 s, 55 °C for 30 s, and 68 °C for 4 min; followed by 68 °C for 10 min. The nested PCR (20 μL total reaction volume) was performed under the same cycling conditions using primers EnvA (5'-GGCTTAGGCATCTCCTATGGCAGGAAGAA-3') and EnvN (5'-CTGCCAATCAGGGAAGTAGCCTTGTGT-3'). Amplicons were visualized on 1% agarose gels, and wells yielding ~3 kb *env* products were recorded. Products from positive wells were purified and submitted for Oxford Nanopore long-read sequencing. Briefly, HIV-1 *env* amplicons were processed by the Ligation Sequencing Kit V14 and PromethION 4.10.4.1 Flow cells using super accuracy base calling models to generate precise linear consensus sequences from raw reads. The orientation of the linear sequences was corrected by aligning to HXB2 in Geneious Prime. Consensus sequences of individual *env* variants were assembled using multiple SGA-derived *env* sequences for each culture well.

Calculation of instantaneous inhibitory potential (IIP) values

Standard dose-response curves show the fraction of infection events that are unaffected by an antiviral agent (*fu*) and typically decrease as the drug concentration increases (Supplementary Fig. 3A). While antiviral agents may have identical IC_{50} values, the slope of the dose-response curve significantly determines *in vivo* efficacy and clinical outcomes[11,12,13,14]. By transforming the linear-log dose-response curve into a log-log plot, inhibition of infection at physiologic concentrations can be most simply expressed as the IIP, the log reduction in infection events (Supplementary Fig. 3B)[11,12,13,14]:

$$IIP = \log_{10}\left(\frac{1}{f_u}\right) = \left(1 + \left(\frac{C}{IC_{50}}\right)^m\right)$$

Dose-response curves can be linearised using the median-effect equation:

$$\log_{10}\left(\frac{1 - f_u}{f_u}\right) = m\log_{10} C - m\log_{10} IC_{50}$$

where C is the *in vivo* concentration of an antiviral agent (e.g., antiretroviral drugs or autologous IgG) in plasma or extracellular fluid, and m is the slope parameter or Hill coefficient (Supplementary Fig. 3C).

Previous studies have demonstrated that individual drugs in modern ART regimens typically have IIP values <3 at normal plasma concentrations[11,12,13,14]. This is insufficient clinically to prevent viral replication *in vivo*. Moreover, effective suppression of viremia typically requires ART regimens with combined IIP values >5 (Supplementary Fig. 3C). Considering the observed IC_{50} and the slope of the dose-response curve, the number of logs of inhibition of single-round infection that purified autologous IgG will cause at typical physiologic IgG concentrations (6-17 mg/mL) can be estimated. Therefore, IC_{50} and IIP values (at an IgG

concentration of 10 mg/mL; $IIP_{10\ mg/mL}$) were calculated to evaluate autologous neutralising antibody potency and *in vivo* efficacy against reservoir virus in ID107 and ID142.

<u>Immunoassays:</u>

Lymphocyte proliferation assay

PBMCs (1 x 10^6 cells/mL) were washed in PBS and stained with 5µM CellTrace Violet (CTV, ThermoFisher, C34557) for 20 minutes at room temperature. Subsequently, the reaction was quenched by adding 5x the original staining volume cRPMI and incubating for 5 minutes at room temperature. The stained cells were then stimulated for 6 days with one of three different HIV-1 peptide pools (Gag (JPT, PM-HIV-Gag), Nef (JPT, PM-HIV-Nef) and Pol (JPT, PM-HIV-Pol); 1µg/mL), dimethyl sulfoxide (DMSO) for the negative control, or anti-human CD3 and anti-human CD28.2 (Nordic Biosite) (1µg/mL) as a positive control. For each stimulation, 2x10^5 cells were included. On day 6, cells were washed with PBS and stained for viability (LIVE/DEAD Fixable NEAR-IR, Invitrogen) and incubated for 20 minutes. Then, cells were incubated for 10 minutes with Human TruStain FcX (BioLegend, catalogue number 422302) in PBS with 2% FBS, followed by 30 minutes incubation with surface stain (1 µL PE/Dazzle™ 594 anti-human CD3 (OKT3, Biolegend, catalogue number 317345, lot number B443448), 0.5 µL PE/Fire™ 700 anti-human CD4 (SK3, Biolegend, catalogue number 344665, lot number B437043) and 2 µL APC anti-human CD8 (SK1, Biolegend, catalogue number 344721, lot number B441271)) in Brilliant Stain Buffer Plus (BD Horizon, catalogue number 566385). The samples were run on MACSQuant16 (Miltenyi Biotec, 130-109-803), and flow data was analysed using FlowJo™ v10.10.0 Software (BD Biosciences). Percentage

proliferating cells was gated as populations dimmer than the undivided peak (Supplementary Fig. 6) with percentage proliferation of negative control subtracted.

IFN-γ enzyme-linked immunosorbent spot assay (ELISPOT)

ELISPOT was performed as described previously[3]. In brief, after 3 hours rest, cells were washed and plated at $1x10^5$ cells per well in Multiscreen 96 well plates (Millipore, MSIPS4W10) coated with unconjugated anti-human IFN-γ mAb (1-D1K) (Mabtech, 3420-3-1000) and stimulated for overnight incubation (~18 hours). For each participant sample, 8 stimulations were applied; 3 negative controls (DMSO), one of three different HIV-1 peptide pools as described above for the lymphocyte proliferation assay (HIV-Gag, HIV-Nef, HIV-Pol, 1 μg/mL), and 2 positive controls (CEF (JPT, PM-CEF-S-3, 1 μg/mL) and staphylococcal enteroxin B (SEB, 1 μg/mL)). The following day, plates were washed and incubated with anti-human IFN-γ mAb (7-B6-1) conjugated to biotin (Mabtech, 3420-6-250) for 1 hour at room temperature. Plates were then washed and subjected to 1 hour incubation with streptavidin-ALP enzyme conjugate (Mabtech, 3310-10-1000). Finally, spots were developed by the AP conjugate substrate kit (Biorad, 1706432) and read using the CTL Immunospot reader (S6 Entry M2, Cellular Technology Ltd., Cleveland, OH, USA). The response was quantified as spot-forming cells (SFC) per million input PBMCs. A positive response was defined as the response minus whichever of the following three background criteria were the highest; at least five spots per well, mean number of spots in negative-control wells plus three times standard deviation and three times the mean of negative-control wells[3].

Activation-induced marker (AIM) assay:

The AIM assay was performed for PBMCs sourced from ID107 and ID142 as previously described[3]. Briefly, following the 3hr rest step, 1 x 10^6 cells were plated into wells within a 96-well plate. Cells were stimulated with each of the following conditions: no exogenous stimulation (DMSO) as negative control, one of three different peptide pools as described for the lymphocyte proliferation and ELISPOT assays (HIV-Gag, HIV-Nef and HIV-Pol, as well as HIV-Env; 2 μg/mL), and SEB (1 μg/mL) as positive control. Cells were incubated with the relevant stimuli for 20 hours at 37°C, and were then washed with PBS and stained for viability with Near IR Live Dead (Invitogen, L10119) for 20 min. Cells were then incubated with Human TruStain FcX (BioLegend, 422301) in PBS + 2% FBS for 10 min, and then were stained for 30 min with the following surface marker antibodies: CD3 (1 μL PerCP/Cy5.5 anti-human CD3, SK7, BioLegend, catalogue number 300429, lot number B354280), CD4 (2 μL, BV650 anti-human CD4, RPA-T4, BioLegend, catalogue number 300535, lot number B366745), CD8 (1 μL, BV605 anti-human CD8a, RPA-T8, BioLegend, catalogue number 301039, lot number B435856), 4-1BB (2.5 μL, PE anti-human CD137, BioLegend, catalogue number 309803, lot number B367290), CD69 (1 μL, APC anti-human CD69, FN50, BioLegend, catalogue number 310909, lot number B359249) and PD-L1 (2.5 μL, BV421 anti-human CD274, B7-H1, BioLegend, catalogue number 374507, lot number B388684). Cells were then washed twice and acquired on a MACS Quant Analyser 16. Data were analysed using FlowJo 10.10.0 (Supplementary Fig. 7). The frequency of antigen-specific cells (AIM+ cells) from each stimulus was determined by subtracting the frequency of the negative control condition from each antigen stimulated condition (GAG, NEF or POL). AIM+ cells were defined as those that co-expressed two or more of the following markers: CD69, PD-L1, or 4-1BB. Total HIV-1-

specific AIM+ cells was calculated as summation of each of the three independent antigen stimulations (GAG + NEF + POL).

For PBMCs sourced from ID9254, the AIM assay was performed as described by Niessl et al.[15]. Briefly, PBMCs were thawed and rested for 3hr at a concentration of 10 x 10^6 cells/mL in 24-well plates, using culture media consisting of RPMI 1640 supplemented with HEPES, penicillin and streptomycin and 10% human serum (Sigma). Cells were then stimulated for 18hr with peptide pools representing Gag, Pol, gp120, gp41 and Nef (NIH AIDS Reagent Program) or DMSO. Following stimulation, cells were stained for viability (aquavivid, Life Technologies) and for surface markers for 30 min at 4°C using the following antibodies: CD3 (3 µL PerCP/eFluor710 anti-human CD3, SK7, eBioscience, catalogue number 46-0036, lot number 1941534), CD4 (4 µL, BUV496 anti-human CD4, SK3, BD Biosciences, catalogue number 564651, lot number 9080989), CD8 (2 µL, BV711 anti-human CD8a, RPA-T8, BioLegend, catalogue number 301044, lot number B237121), 4-1BB (5 µL, PE-Cy7 anti-human CD137, 4B4-1, BioLegend, catalogue number 309818, lot number B258325), CD69 (5 µL, BUV395 anti-human CD69, FN50, BD Biosciences, catalogue number 564364, lot number 8242749) and PD-L1 (5 µL, BV421 anti-human CD274, 29E2A3, BioLegend, catalogue number 329714, lot number B258010). Cells were then fixed using 2% paraformaldehyde, and then were acquired using the LSRII flow cytometer (BD Biosciences). Data were analysed using FlowJo v.10.5.0. The gating strategy can be found in Niessl et al.[15].

Spectral flow cytometry intracellular cytokine staining (ICS): post-acquisition analysis

Data preprocessing and quality control

Spectral unmixing was performed using the ID7000 Software version 2.0.2 (SONY Biotechnologies, San Jose, CA) utilising the Weighted Least Squares Method (WLSM) algorithm. All analyses downstream of spectral unmixing were conducted in R version 4.3.2 with RStudio version 2023.12.1.402 using an in-house pipeline built through the assembly of several algorithms. Unmixed FCS-files from the ID7000 Software were read in R as a *flowSet* class and transformed using estimateLogicle transformation from flowCore v2.20.0[16]. Transformed data was pregated as a *gatingSet* class gating on cells, live, singlets, lymphocytes, CD16-CD19-, CD3+ lymphocytes using ggcyto v1.36.1[17] (Supplementary Fig. 8). Quality control was performed on pre-gated data using the R implementation of PeacoQC v1.18.0[18] with MAD = 4 and IT = 0.1.

Clustering and dimensionality reduction

The cleaned *flowSet* class data was converted to a *single cell experiment (SCE)* class using the CATALYST package v1.32.1[19]. FlowSOM (v2.16.0) clustering and ConsensusClusterPlus metaclustering[20] was performed using CATALYST's (v1.32.1) *cluster* function with a SOM grid size of 10. Cells were visualized on UMAPs composed of 10,000 cells per sample usings CATALYST *runDR* function with n_neightbours = 15 and min_dist = 0.01. Markers used to build UMAPs were the same as those used for clustering.

First, the lymphocyte population was clustered into main populations using canonical lineage markers (CD3, CD8, CD4, CD16, CD19). A resolution giving 15 metaclusters was used, revealing NK cells, B cells, CD4 T-cells, CD8 T-cells, CD4-CD8- T-cells, CD4+CD8+ T-cells, and lineage negative (CD3-CD16-CD19-) lymphocytes (Supplementary Fig. 9).

CD4 and CD8 T-cells were further subclustered separately using a combination of the top 10 variable markers defined by their PCA-based non-redundancy score (NRS) and markers chosen based on prior knowledge of their biological relevance for certain cell types (e.g. FoxP3 for regulatory CD4 T-cells). For CD4 T-cells, 12 markers were used (CD45RA, CD95, CCR7, CD27, TCF1, CCR4, TIGIT, CD39, FoxP3, PD-1, CXCR5, Tbet), and for CD8 T-cells, 11 markers were used (CD45RA, CD27, TIGIT, TCF1, CD95, CCR7, CD16, Tbet, CD8, PD-1, CCR4).

Memory T-cell polyfunctionality

Memory T-cell clusters were identified by their expression of CD45RA, CCR7, CD27, and CD95 (Supplementary Fig. 10-11). Cells were gated for the degranulation marker CD107a and the cytokines IFN-γ, TNF-α, and IL-2 in *R* using ggcyto (Supplementary Fig. 12). The percentage of cells positive for each of the fifteen combinations of markers was calculated and cluster-wise (i.e. CD4 and CD8 memory clusters) background subtraction was performed to define peptide-specific signal. We defined polyfunctionality as cells positive for two or more markers. To compare the frequencies of mono- and polyfunctional memory CD4 and CD8 T cells between PICs and ART controls, two-sided, unpaired, non-parametric Wilcoxon signed rank tests with a Bonferroni correction for multiple comparisons were used.

PDX mouse model – Detailed methodology

mCD4 T-cells were isolated from PBMCs sourced from ID107 ATI week 53 by negative selection (STEMCELL, 19157), and NSG female mice were engrafted via i.v. injection with 5×10^{6} memory CD4 (mCD4) T-cells resuspended in 100 µl Hank's Balanced Salt Solution

(HBSS) (Gibco, 14025092). Three weeks post-mCD4 T-cell engraftment, the mice were bled weekly to quantify human cell counts and viral load. Approximately 100 µl of peripheral blood was collected into EDTA-coated tubes (Kent Scientific, MCVT100-EDTA) via tail vein nick technique and processed immediately. Blood was centrifuged at 5000xg for 5 minutes to separate plasma and stored at -80°C until viral RNA extraction. Cell counts were calculated using CountBright Absolute Counting Beads (Invitrogen, C36950) and analysed by flow cytometry (Attune NxT, Invitrogen, A24858). Spontaneous viral rebound occurred in two mice and therefore plasma from these two viremic mice was used to infect the remaining mice. Mice were infected via i.p. injection with autologous virus diluted to a total of 100 µl HBSS. A week following two rounds of infection, mice were divided across two groups for equivalent CD4 T-cell counts and viral loads (Supplementary Table 10). One group of mice were engrafted via i.v. injection with $5x10^6$ autologous mCD8 T-cells resuspended in 100 µl HBSS (+ CD8 T-cell group). mCD8 T-cells were isolated from PBMCs from the same timepoint through negative magnetic selection (STEMCELL, 19159).

Viral load was quantified weekly by measuring HIV RNA concentrations in plasma using the integrase single-copy assay previously described[21]. Briefly, RNA was isolated from plasma using the QIAamp Viral RNA Mini Kit (QIAGEN, 52906) and the QIA cube HT system (QIAGEN, 9001896) and subsequently stored at -80°C. The qPCR master mix was prepared using the AgPath-ID One-Step RT-PCR reagents (Thermo Scientific, 4387391) and the qPCR was carried out using a QuantStudio 7 Pro Real-Time PCR system (Applied Biosystems, A43183) under the following conditions: 45°C for 10 min, 95°C for 10 min, followed by 40 cycles at 95°C for 15 s and 60°C for 1 min. Primer and probe sequences were as follows: forward

primer: 5’-TTTGGAAAGGACCAGCAAA-3’; reverse primer: 5’-CCTGCCATCTGTTTTCCA-3’; 5’-6FAM-AAAGGTGAAGGGGCAGTAGTAATACA-BHQ1-3’[22]. To determine the HIV RNA concentration, cycle threshold values were compared with a validated HIV RNA standard.

Check of unique viral variant for possible superinfection following viral rebound in ID142:

To check for the possibility of superinfection with a genetically unrelated viral strain causing the viral rebound in ID142, two analyses were performed. First, all genetically intact proviral, qVOA and plasma-derived sequences obtained from pre- and post-rebound timepoints were aligned with the HIV-1 subtype B lab strain sequences HXB2 (accession no. K03455) and NL4-3 (accession number AF324493.2), trimmed to the ~8.7 kb region sequenced by PRLS. Phylogenetic comparisons were performed using PhyML[23], using the HKY85 nucleotide substitution model and a gamma rate of 4. Branch support was inferred using 1,000 bootstraps. Phylogenetic trees were visualised using ggTree[24].

Second, HIV-1 subtype B *env* sequences were obtained from six individuals previously sequenced as part of the eCLEAR and TITAN studies, including 20-30 sequences per individual[10,25]. All ID142 genetically intact sequences were trimmed to *env*, and phylogenetic comparisons were performed as described above.

Supplementary Table 1 – Clinical characteristics of PICs

**Pre-ART clinical characteristics**	**ID107**	**ID142**	**ID9254**
Age	45	57	48*
Sex	Male	Male	Male
Ethnicity	White or Caucasian	White or Caucasian	White or Caucasian
Viral load	188,945 copies/mL	215,805 copies/mL	860,000 copies/mL
CD4 count	470 cells/mm^3	590 cells/mm^3	750 cells/mm^3
Timing of ART initiation post-HIV-1 acquisition	2 months	5 months	4-5 months (estimated)
ART regimen	TDF, FTC, DTG	TAF, FTC, BIC	EVG, cobi, TAF, FTC*
HIV-1 subtype/circulating recombinant form	CRF01	B	B
HLA-A	02:01:01/25:01:01	01:01:01/02:01:01	01/29
HLA-B	15:01:01/44:02:01	08:01:01/44:02:01	38/44
HLA-C	03:03:01/05:01:01	05:01:01/07:01:01	12/16
HLA-DR	04:01:01/13:01:01	03:03:01/04:04:01	
HLA-DQ	03:02:01/06:03:01	02:01:01/03:02:01	
HLA-DP	02:01:02/19:01:01	03:01:01/04:01:01	
Clinical trial (arm)	eCLEAR (3BNC117 + RMD)	TITAN (3BNC117/10-1074 + placebo)	3BNC117 and 10-1074 infusion during ATI
bNAb sensitivity at enrolment	Sensitive (Phenosense)	Sensitive (Phenosense)	Sensitive (in vitro neutralisation)
Time on ART at ART interruption	57 weeks	287 weeks	1,096 weeks (21 years)
Time off ART	334 weeks (ongoing)	130 weeks	388 weeks (ongoing)

TDF, tenofovir disoproxil fumarate. FTC, emtricitabine. DTG, dolutegravir. TAF, tenofovir alafenamide. BIC, bictegravir. EVG, elvitegravir. cobi, cobicistat.

*at enrollment

Supplementary Table 2. Integration sites of genetically intact proviruses in ID107 and ID142

Chromosome	Position	Gene	Timepoints identified in ATI weeks (no. times identified)
**ID107**			
5	24162002	long non-coding RNA	Week -57 (pre-ART)
			Week 0 (on-ART)
16	89756643	FANCA	Week -57 (pre-ART)
22	27857113	PITPNB	Week -57 (pre-ART)
17	59099605	TRIM37	Week -57 (pre-ART)
19	58232791	ZNF544	Week -57 (pre-ART) (x 2)
19	14156973	ADGRL1	Week -57 (pre-ART)
19	1104407	SMARCA4	Week -57 (pre-ART)
8	7890581	non-genic	Week -57 (pre-ART)
12	67512040	LINC02408	Week -57 (pre-ART)
X	40692154	MED14	Week -57 (pre-ART) (x 2)
15	65754467	DENND4A	Week -57 (pre-ART)
18	12370315	CHRDL2	Week -57 (pre-ART)
7	37230260	ELMO1	Week -57 (pre-ART) (x 2)
19	36579961	ZNF529	Week -57 (pre-ART) (x 2)
14	74673138	AREL1	Week -57 (pre-ART) (x 2)
17	59580235	DHX40	Week -57 (pre-ART) (x 2)
22	12100932	non-genic; large gene desert	Week 29
			Week 281
Y	11286950	non-genic; pericentromeric	Week 130 (x 4)
			Week 227 (x 2)
			Week 281 (x 2)
4	49103577	centromere	Week 130 (x 8)
			Week 227
			Week 281 (x 4)
19	24264777	centromere	Week 227
			Week 281 (x 5)
X	96989587	DIAPH2	Week 227
9	43319381	centromere	Week 227
4	49633350	centromere	Week 281
**ID142**			
21	8471352	non-genic; large gene desert	Week -2 (x 4)
			Week 78

Supplementary Table 3. Neutralisation capacity of IgG from ID107 against global virus panel.

Virus ID	Clade	IgG - ATI week 29 (IC_{50})	IgG – ATI week 181 (IC_{50})	IgG – ATI week 255 (IC_{50})
ID107 qVOA.1	Autologous	0.838	1.305	0.882
R1166.c01	CRF01_AE	>500	>500	>500
C1080.c03	CRF01_AE	>500	>500	>500
C3347.c11	CRF01_AE	>500	>500	>500
CNE5	CRF01_AE	>500	>500	>500
C4118.c09	CRF01_AE	>500	>500	>500
R2184.c04	CRF01_AE	>500	>500	>500
R3265.c06	CRF01_AE	>500	>500	>500
C2101.c01	CRF01_AE	>500	>500	>500
Q259.d2.17	A	>500	>500	>500
Q461.e2	A	>500	>500	>500
Q769.d22	A	>500	>500	>500
3415.v1.c1	A	>500	>500	>500
6535.3	B	>500	>500	>500
SC422661.8	B	>500	>500	>500
REJO4541.67	B	>500	>500	>500
CAAN5342.A2	B	>500	>500	>500
ZM135M.PL10a	C	>500	>500	>500
ZM53M.PB12	C	>500	>500	>500
BF1266.431a	C	>500	>500	>500
Ce1176_A3	C	>500	>500	>500
AC10.0.29	B	>500	>500	>500
ZM247v1 (Rev-)	C	>500	>500	>500
620345.c01	CRF01_AE	>500	>500	>500

Supplementary Table 4. Neutralisation capacity of IgG from ID9254 against global virus panel.

**Virus ID**	**Clade**	**IgG – Pre-infusion (IC_{50})**	**IgG – ATI week 42 (IC_{50})**
Q769.d22	A	>500	>500
TRO.11	B	>500	>330
Q259.d2.17	A	>500	>330
25710-2.43	C	>500	>330
YU2.DG	B	>500	>330
ZM135M.PL10a	C	>500	>500
CNE8	CRF01_AE	>500	>500
Q842.d12	A	>500	>500
X1632_S2_B10	G	>500	>500
Ce1176_A3	C	>500	>500
246-F3_C10_2	AC	>500	>500
CNE55	CRF01_AE	>500	>500

Supplementary Table 5. Clinical characteristics of non-controller/ART-suppressed participants

**Clinical characteristics**	**ID104**	**ID112**	**ID110**	**ID702**	**ID120**	**ID209**
Age at baseline	32	37	49	33	30	30
Sex	Male	Male	Male	Male	Male	Female
Ethnicity	White or Caucasian	White or Caucasian	White or Caucasian	Black or African European	Asian	White or Caucasian
Timing of ART initiation post-acquisition	2.25 months	7-8 months	0-3 months	4-6 months	0-3 months	0-3 months
ART regimen	TDF, 3TC, DTG	TDF, 3TC, DTG	TDF, 3TC, DTG	ABA, 3TC, DTG	FTC, 3TC, DTG	TDF, 3TC, DTG
HIV-1 subtype	B	G	D	D	B	B
eCLEAR study arm	placebo + romidepsin	3BNC117 + romidepsin	Placebo + placebo	Placebo + placebo	Placebo + placebo	Placebo + placebo
Time on ART at ART interruption	12 months	12 months	12 months	12 months	12 months	12 months
Time to loss of virological control*	35 days	21 days	N/A	34 days	93 days^	N/A
Viral load at ART restart (HIV-1 RNA copies/mL)	6,040	39,800	N/A	54,000	465	N/A
HLA-A	02:01:01/02:01:01	02:01:01/68:01:02	01:01:01/29:02:01	29:02:01/66:01:01	02:03:01/02:03:01	03:01:01/30:04:01
HLA-B	15:01:01/44:05:01	15:78:01/44:02:01	08:01:01/44:03:01	18:01:01/35:01:01	46:01:01/51:01:02	14:02:01/40:02:01
HLA-C	02:02:02/03:28	03:04:01/07:04:01	07:01:01/16:01:01	04:01:01/05:01:01	01:02:01/14:02:01	02:02:02/08:02:01
**Sampling included in study**						
**Pre-ART**						
Assay	AIM-sorting + scRNA-seq and TCR sequencing	AIM-sorting + scRNA-seq and TCR sequencing	None	None	None	None
Viral load (HIV-1 RNA copies/mL)	116,000	23,100	88,000	110,000	15,500	16,900
CD4 count (cells/mm^3)	600	560	1,440	338	580	948
**Pre-ATI**						
Assay	AIM-sorting + scRNA-seq and TCR	AIM-sorting + scRNA-seq and TCR	ICS spectral flow	ICS spectral flow	ICS spectral flow	ICS spectral flow

	sequencing; ICS spectral flow	sequencing; ICS spectral flow				
Viral load (HIV-1 RNA copies/mL)	<20	<20	<20	<20	<20	<20
CD4 count (cells/mm^3)	530	570	1,780	668	1,190	1,430
**Viral rebound**						
Assay	AIM-sorting + scRNA-seq and TCR sequencing; ICS spectral flow	AIM-sorting + scRNA-seq and TCR sequencing; ICS spectral flow	None	None	None	None
Viral load (HIV-1 RNA copies/mL)	6,040	39,800	N/A	54,000	465	N/A
CD4 count (cells/mm^3)	640	790	N/A	377	770	N/A

TDF, tenofovir disoproxil fumarate. 3TC, lamivudine. FTC, emtricitabine. DTG, dolutegravir. ABA, abacavir. TAF, tenofovir alafenamide. BIC, bictegravir. EVG, elvitegravir. cobi, cobicistat.

* Defined as two measurements of plasma viral load >5,000 copies/mL

^Did not reach loss of virological control within ATI period

Supplementary Table 6. Sensitivity of ID9254 Q^2VOA isolates to 3BNC117 and 10-1074

Virus ID	IgG Titre in TZM-bl cells (μg/mL)			
	3BNC117		10-1074	
	IC_{50}	IC_{80}	IC_{50}	IC_{80}
9254_W102_a3	0.225	0.496	0.163	0.377
9254_W102_a20	0.232	0.619	0.158	0.375
9254_W102_a24	0.248	0.648	0.160	0.459
9254_W102_b10	0.232	0.630	0.152	0.457
9254_W102_c2	0.211	0.602	0.136	0.351
9254_W102_c11	0.229	0.547	0.133	0.309
9254_W102_c14	0.219	0.725	0.146	0.416
9254_W102_c15	0.198	0.547	0.157	0.478
9254_W102_c21	0.216	0.564	0.148	0.504
9254_W102_e3	0.217	0.719	0.119	0.335
9254_W102_e8	0.155	0.486	0.080	0.283
9254_W102_e18	0.194	0.467	0.123	0.314
9254_W102_e20	0.195	0.541	0.121	0.375
9254_W102_f4	0.230	0.643	0.128	0.313
9254_W102_f6	0.307	0.670	0.156	0.379
9254_W102_f11	0.236	0.646	0.134	0.315
9254_W102_g1	0.159	0.455	0.101	0.298
9254_W102_g8	0.131	0.385	0.096	0.239
9254_W102_g14	0.275	0.759	0.177	0.411

Supplementary Table 7 – IPDA primer and probe sets

Target	Primer/probe name	Sequence	Citation
HIV-1 Ψ	Ψ forward primer	5'-CAGGACTCGGCTTGCTGAAG-3	[1]
	Ψ reverse primer	5'-GCACCCATCTCTCTCCTTCTAGC-3'	
	Ψ probe	5'-FAM-TTTTGGCGTACTCACCAGT-MGBNFQ-3'	
HIV-1 *env*	secondary *env* forward primer	5'-ACTATGGGCGCAGCGTC-3'	[26]
	secondary *env* reverse primer	5'-CCCCAGACTGTGAGTTGCA-3'	
	secondary *env* probe	5'-VIC-CTGGCCTGTACCGTCAG-MGBNFQ-3'	
RPP30	*RPP30* forward primer	5'-GATTTGGACCTGCGAGCG-3'	[1]
	RPP30 reverse primer	5'-GCGGCTGTCTCCACAAGT-3'	
	RPP30 probe	5'-VIC-CTGACCTGAAGGCTCT-MGBNFQ-3'	
RPP30 shear	*RPP30* shear forward primer	5'-CCATTTGCTGCTCCTTGGG-3'	[1]
	RPP30 shear reverse primer	5'-CATGCAAAGGAGGAAGCCG-3'	
	RPP30 shear probe	5'-FAM-AAGGAGCAAGGTTCTATTGTAG-MGBNFQ-3'	

Supplementary Table 8. Primer sequences for residual viremia sequencing

Primer name	Target	Sequence (5’-3’)	Citation
*env*_FO_ID107	Outer PCR forward	GCCAGTGGTRTCAACYCAA	Modified from[27] to be specific for virus from ID107
*env*_RO	Outer PCR reverse	GCARATGAGTTTTCYAGAGCA	[27]
*env*_FN	Inner PCR forward	CTGCTAAATGGCAGTCTAGC	[27]
*env*_RN	Inner PCR reverse	TTGCCTGGAGCTGYTTRATGC	[27]

Supplementary Table 9 – Antibodies used in ICS-spectral flow assay

Antibody	Fluorochrome	Supplier	Catalogue number	Clone	Lot number	Volume (µL)
Anti-IL-2	BUV395	eBioscience	363-7029-42	MQ1-17H12	2851058	0.5
Anti-CD4	BUV496	BD Bioscience	612936	SK3	3247822, 4204806	2.5
Anti-CD16	BUV563	BD Bioscience	568289	3G8	4095102, 4124989, 4185605	2.5
Anti-CD3	BUV615	BD Bioscience	751252	SK7	4243045, 4243046, 4243047	1
Anti-CXCR5	BUV661	BD Bioscience	741559	RF8B2	5058675	0.04
Anti-Tbet	BUV737	BD Bioscience	568166	O4-46	4102178, 4178340	2.5
anti- IFN-γ	BV421	BD Bioscience	562988	B27	4234096	0.1
Anti-CCR4	BV510	Biolegend	359416	L291H4	B377907	5
Anti-CD19	BV570	Biolegend	302236	HIB19	B386458	5
anti- TNF-α	BV605	Biolegend	502936	Mab11	B422747	2
Anti-CD27	BV650	Biolegend	302828	O323	B375145, B412778	4
Anti-PD-1	BV711	Biolegend	329928	EH12.2H7	B410254	5
Anti-CCR7	BV785	Biolegend	353230	G043H7	B415814	5
Anti-granulysin	AF488	BD Bioscience	558254	RB1	333150	3
Anti-CD8	Sparkblue-574	Biolegend	344786	SK1	B430783	5
Anti-granzyme B	PerCP	Biolegend	396416	QA18A28	B429745	5
Anti-TCF1	Realblue705	BD Bioscience	570635	S33-966	418397	1.25
anti-CD45RA	Realblue780	BD Bioscience	569081	HI100	3341167, 4151115	1
Anti-IL-4	PE	Biolegend	500810	MP4-25D2	255580	0.4
Anti-IL-13	PE	Biolegend	501903	JES10-5A2	3109515, B380784	1
Anti-Ki67	Pe-dazzle594	Biolegend	350534	Ki67	B376072	0.6
Anti-CD95	PE-fire640	Biolegend	305658	DX2	B420907	2.5
Anti-granzyme K	PE-Cy7	Biolegend	370516	GM26E7	B424505	10
Anti-CD39	Pe-fire810	Biolegend	328245	A1	B427223	2.5
Anti-CD107a	APC	Miltenyi	130-119-869	H4A3	5240808332	5
Anti-FoxP3	SparkNIR-685	Biolegend	320130	206D	B42255	1.5
Anti-TIGIT	R718	BD Bioscience	569038	TgMab-2	4304734	3
Anti-perforin	APC-fire750	Biolegend	353318	B-D48	B400537	5

Supplementary Table 10. HIV-1 viral load and CD4 T-cell count for PDX mice

Group	No. mice	CD4 T-cell count (cells/µL) (mean, SD)	Viral load (HIV-1 RNA copies/mL) (mean, SD)
- CD8 T-cells	12	145, 123	3.71×10^6, 2.54×10^6
+ CD8 T-cells	7	122, 116	5.15×10^6, 4.45×10^6

Supplementary Table 11. Clinical characteristics of non-controller individual included in PDX mouse experiment

**Sex**	Female
**Age**	52 years old
**Ethnicity**	African American
**ART initiation**	Early post-acquisition
**Viral load (at ART initiation)**	1,366 copies/mL
**Nadir CD4 T-cell count**	1,254 cells/mL
**CD4/CD8 ratio**	1.04
**HIV-1 reservoir size**	735 HIV-1 Gag copies/10^6 CD4 T-cells
**Inducible HIV-1 reservoir size (qVOA)**	3.59 IUPM

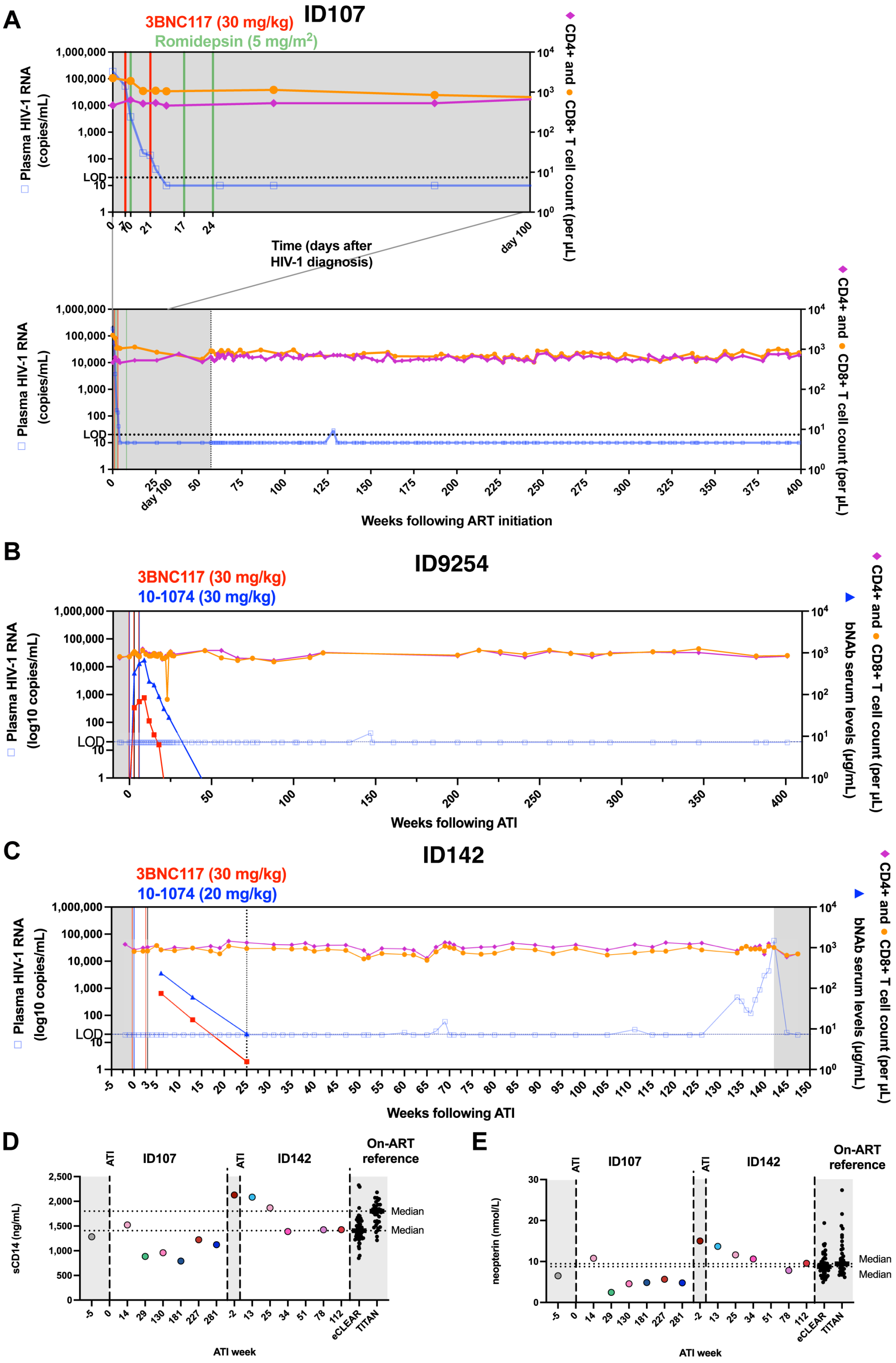

Supplementary Fig. 1. Clinical measures through time on- and off-ART. CD4 and CD8 T-cell counts (right axis) over time following ART initiation compared to plasma HIV-1 RNA viral load (left axis) and CD4 and CD8 T-cell counts (right axis) for ID107 (A). Time of dosing of the

bNAb 3BNC117 (red) and the HDACi romidepsin (green) are shown in the top inset. CD4 and CD8 T-cell counts (right axis) for ID9254 (B) and ID142 (C) compared to plasma HIV-1 RNA viral load (left axis) over time in weeks following ATI. Serum levels of the bNAbs 3BNC117 (red) and 10-1074 (blue) are also indicated. CD4 T-cell counts are represented in pink and CD8 T-cell counts are represented in orange. Limit of detection (LOD) is 20 HIV-1 RNA copies/mL. Grey shading indicates time on ART. Plasma (D) sCD14 and (E) neopterin concentrations were quantified at multiple timepoints for ID107 and ID142 and compared to the levels measured in two cohorts of ART-suppressed individuals[10,25]. Dashed lines represent median values for each of the two cohorts. Grey shading indicates time on ART. Analysed samples are colour-coded according to timepoint.

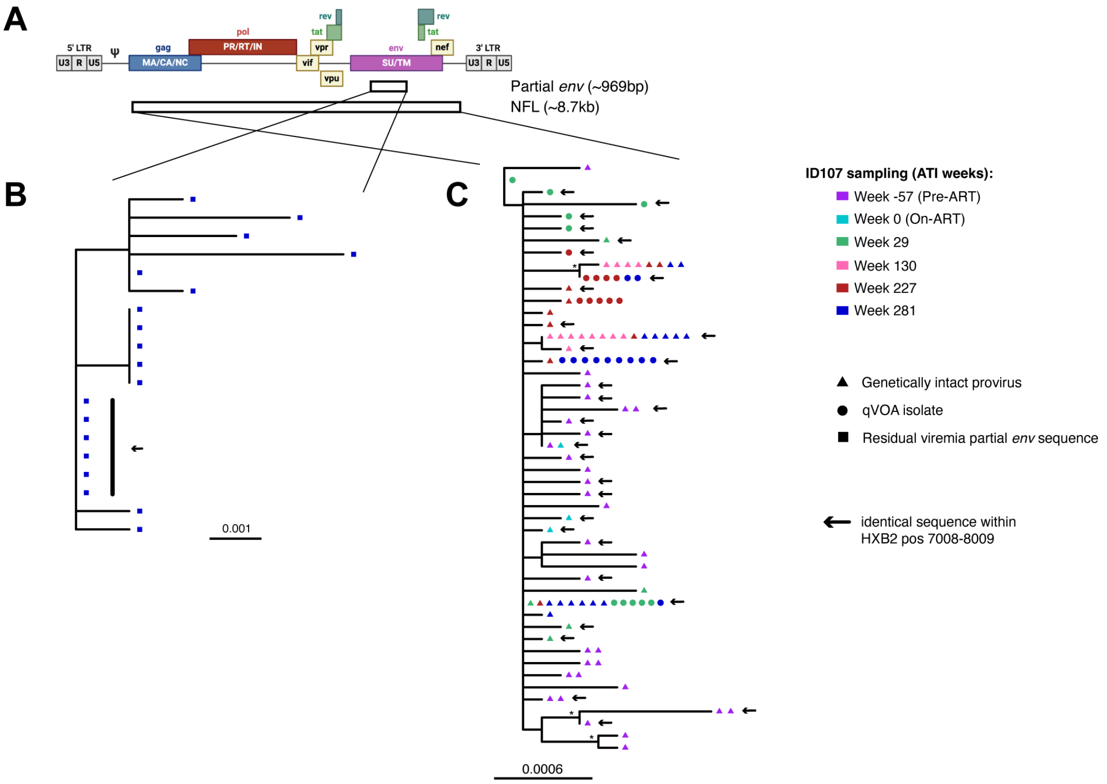

Supplementary Fig. 2. *env* sequences sourced from residual viremia for ID107. (A) Depiction of the HIV-1 genome and the length of amplicons used to sequence and characterise residual viremia (partial env region; ~969bp), and proviral and positive qVOA wells (near-full-length, NFL; ~8.7 kb) in ID107. Created in BioRender (Fisher, K. (2026) https://BioRender.com/agvvcst). (B) ML phylogenetic tree showing all *env* sequences (HXB2 position 7008-8009) sourced from plasma residual viremia from ATI week 281. (C) ML phylogenetic tree showing all genetically intact proviruses isolated by MIP-seq and inducible replication-competent HIV-1 RNA genomes sequenced from positive qVOA cultures, as shown in Figure 2F. Arrows indicate sequences that are genetically identical within the sequenced *env* region to proviral and inducible replication-competent sequences identified with the arrow in (B). Scale bars represent nucleotide substitutions per site. * indicates branch support value >70%.

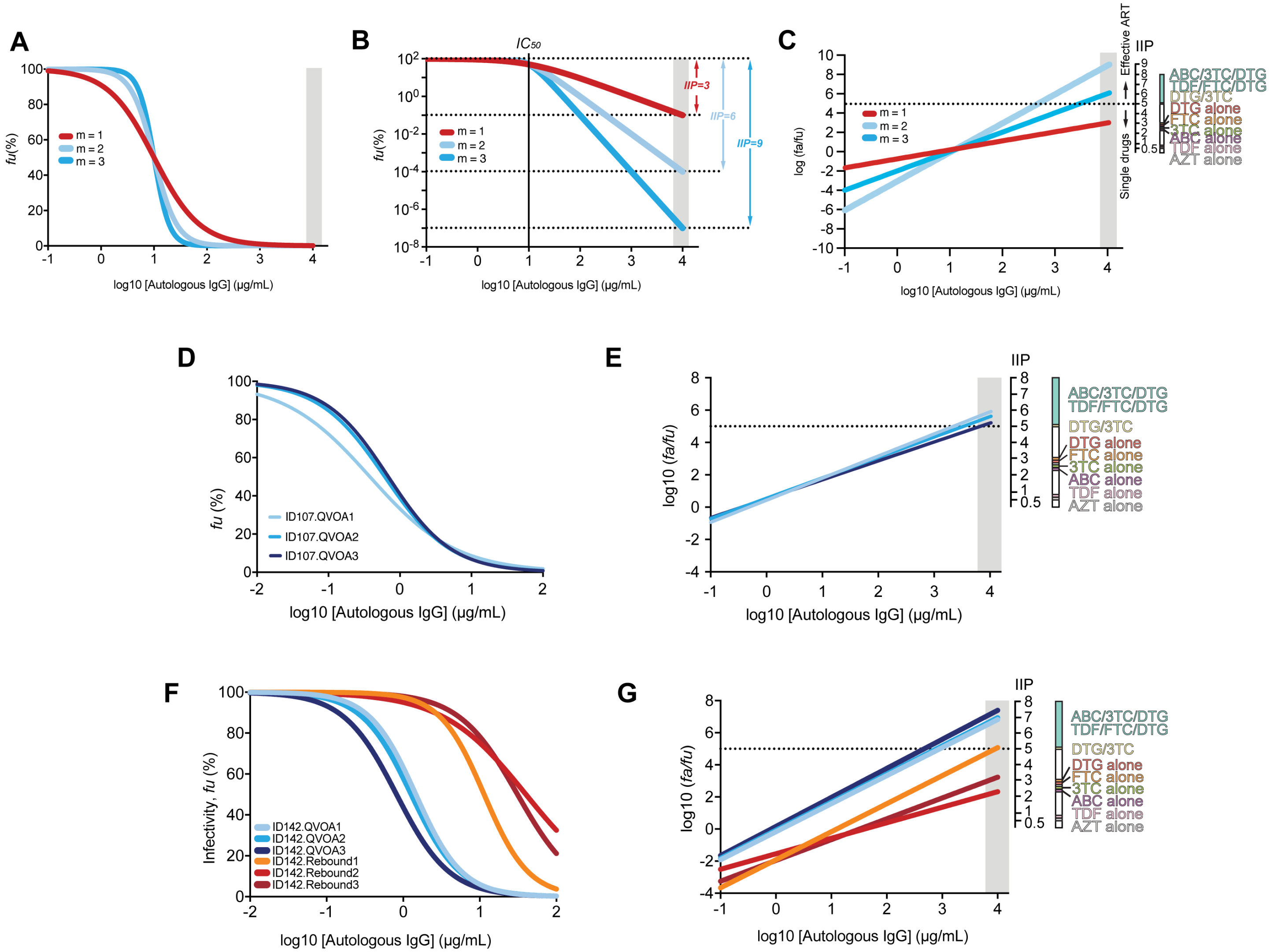

Supplementary Fig. 3. Use of instantaneous inhibitory potential (IIP) to estimate in vivo antiviral activity of aNAbs. (A)

Standard dose-response curves for three hypothetical antiviral agents with the same IC_{50} (10 µg/mL) but different values of the

slope parameter (m, the Hill coefficient). Curves show that the fraction of infection events that are unaffected by the antiviral agent as a percent (fraction unaffected, fu) decreases as the concentration of the antiviral agent increases. (B) Transformation of the linear-log dose-response curve in (A) into a log-log plot shows that inhibition is strongly dependent on the slope parameter. The log reduction in infection events is known as IIP. (C) Dose-response curves in (A) and (B) can be linearized using the median-effect equation (see Supplementary Notes). This allows extrapolation of the inhibitory effect into the clinical concentration range. The right y-axis compares IIP values to the degree of inhibition mediated by average plasma concentrations of single antiretroviral drugs (IIP<5) and effective combination antiretroviral drugs (IIP>5)[11,12]. The grey shaded region represents physiological concentrations of autologous IgG in vivo ranging from 6-17 mg/mL. (D) Dose-response curves for isolates ID107.QVOA1, ID107.QVOA2, and ID107.QVOA3 against autologous IgG purified from ATI week 281. (E) Dose-response curves for ID107 transformed into a median-effect plot. (F) Dose-response curves for isolates ID142.QVOA1, ID142.QVOA2, ID142.QVOA3, ID142.REBOUND1, ID142.REBOUND2 and ID142.REBOUND3 against autologous IgG purified from the time of initial viral rebound at ATI week 130. (G) Dose-response curves for ID142 transformed into a median-effect plot. ABC, abacavir. 3TC, lamivudine. DTG, dolutegravir. TDF, tenofovir disoproxil fumarate. FTC, emtricitabine. AZT, azidothymidine.

A

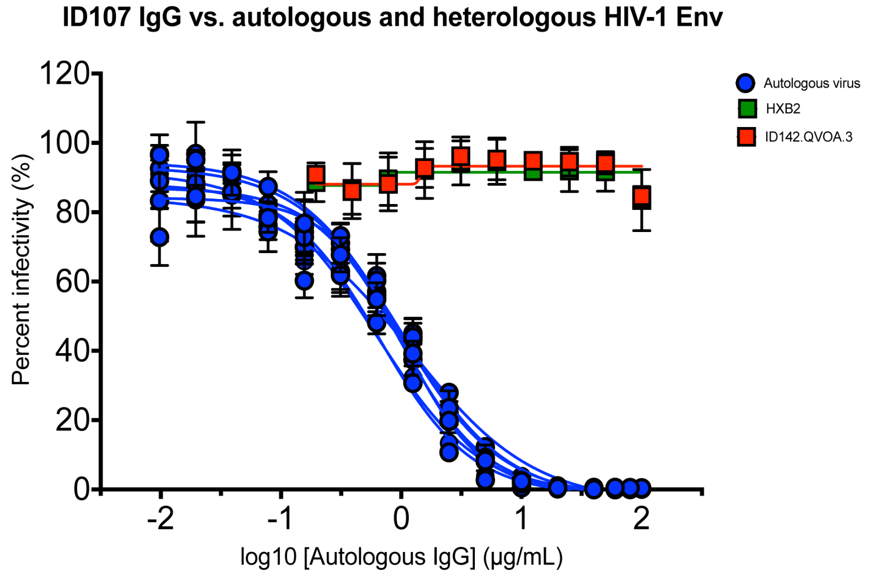

B

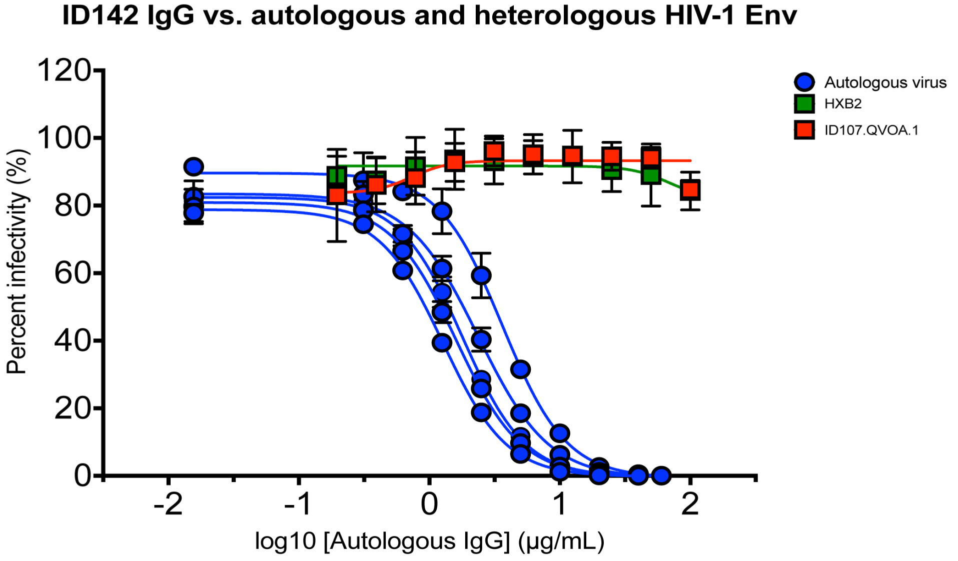

Supplementary Fig. 4. Autologous antibodies from post-intervention controllers potently neutralise autologous virus but not heterologous variants. (A) Neutralisation of pseudoviruses by IgG from ID107 ATI week 281. Autologous ID107 virus (blue) is potently neutralised, whereas the lab-adapted HXB2 pseudovirus (green) and ID142 pseudovirus (red) are not neutralised. (B) Neutralisation of pseudoviruses by IgG from ID142 ATI week 112. Autologous ID142 virus (blue) is potently neutralised, whereas HXB2 (green) and ID107 (red) pseudoviruses are not neutralised. Data representing neutralisation of autologous

pseudoviruses by autologous IgG is identical to that shown in Figures 3C and 3F. Datapoints represent mean ± SD, based on 2-3 technical replicates of culture wells containing pseudovirus and TZM-bl cells for each IgG concentration. For each experiment, control wells were included containing pseudovirus and TZM-bl cells with no IgG (6 wells per experiment) and TZM-bl cells with no pseudovirus or IgG (6 wells per experiment).

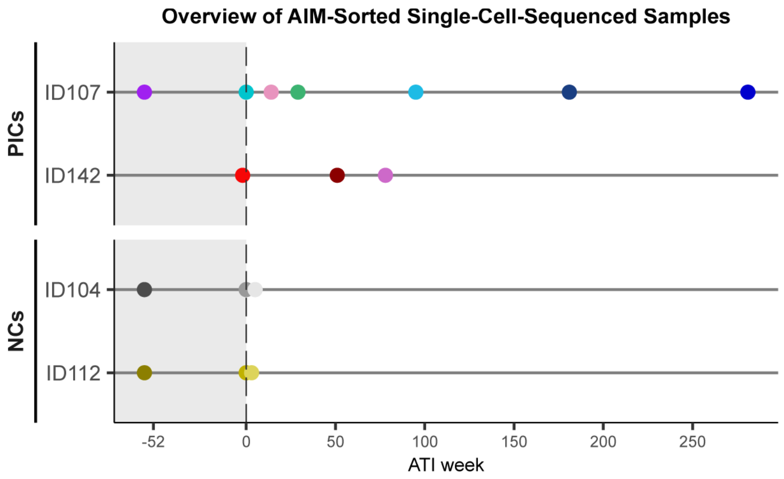

Supplementary Fig. 5. Sampling for single-cell sequencing of AIM+ T-cells. Overview of single-cell sequenced samples analysed per participant and time point. Grey shading represents time on ART. Analysed samples are colour-coded according to timepoint.

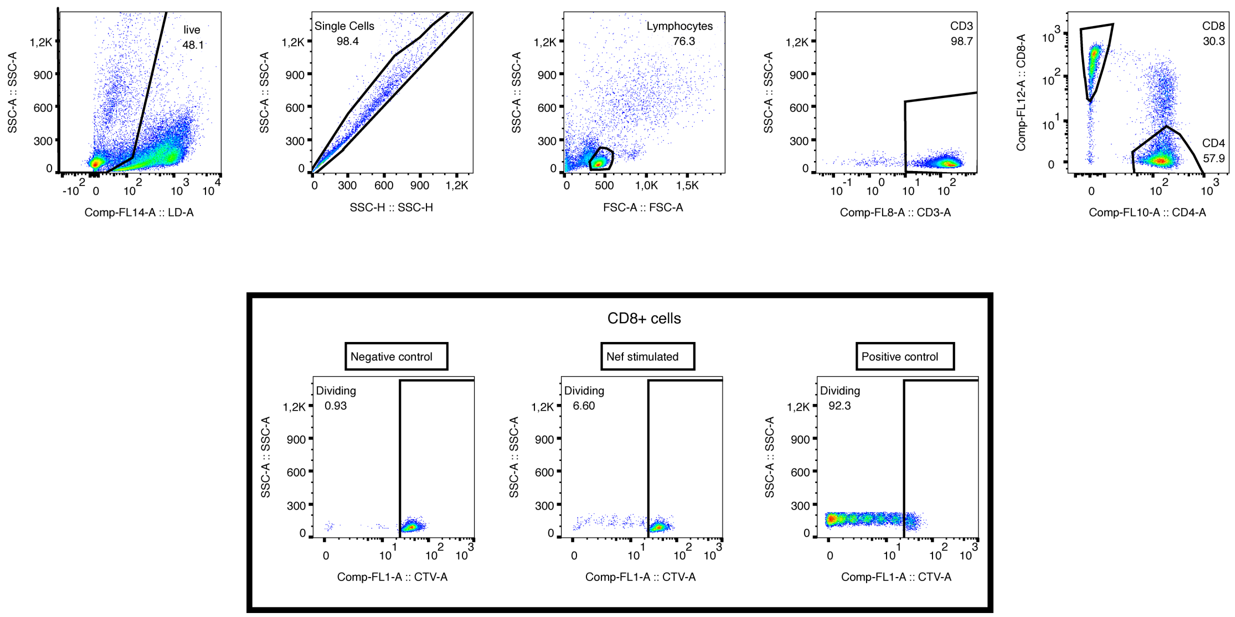

Supplementary Fig. 6. Gating strategy for T-cell proliferation assays. Representative sample. The same gating strategy was used for dividing CD4+ cells, as for CD8+ cells. Figures were created in FlowJo Layout Editor.

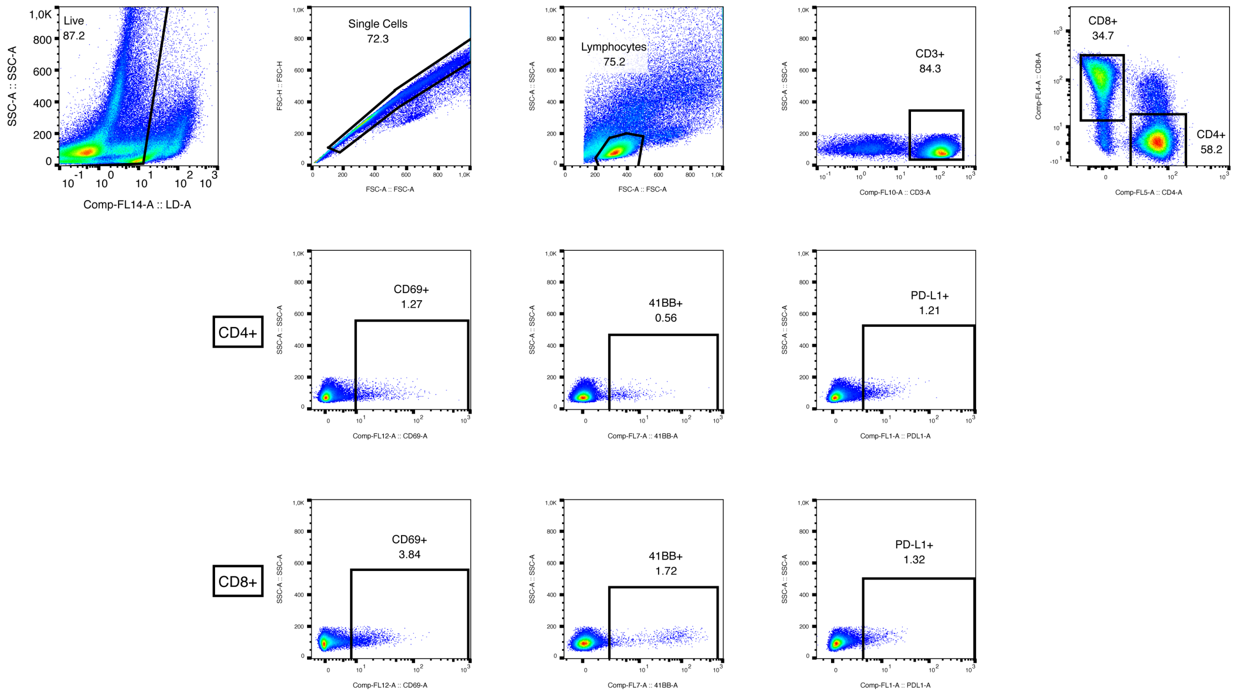

Supplementary Fig. 7. Gating strategy for activation-induced marker (AIM) assay.

Representative sample. Figures were created in FlowJo Layout Editor.

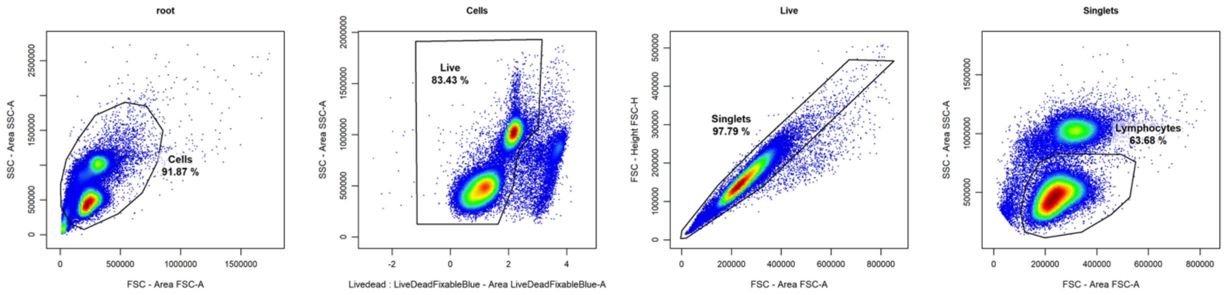

Supplementary Fig. 8. Spectral flow cytometry ICS pre-gating strategy. Representative sample showing the gating strategy for pregating of lymphocytes prior to clustering.

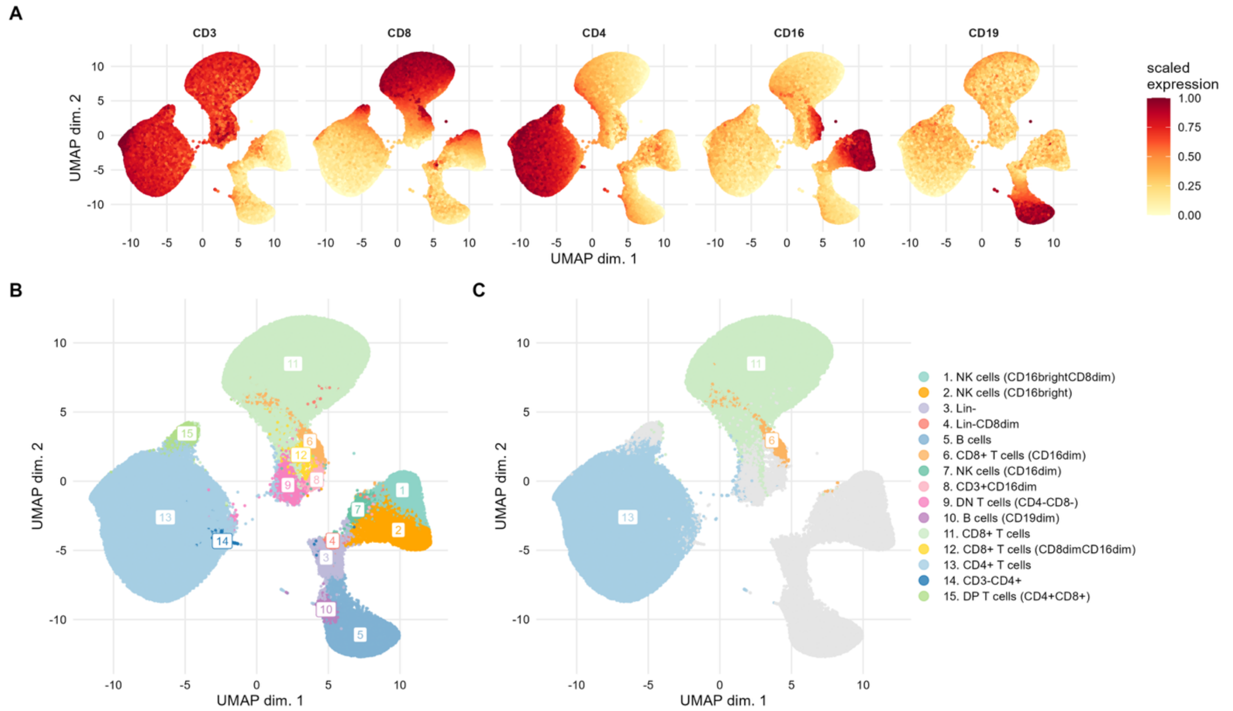

Supplementary Fig. 9. Clustering of main lymphocyte populations in spectral flow cytometry ICS. (A) UMAPs showing expression of markers used for clustering. (B, C) UMAP coloured by FlowSOM cluster labels with clusters 6, 11, 12 (CD8 T-cells) and 13 (CD4 T-cells) highlighted, which are further subclustered (see Supplementary Fig. 10).

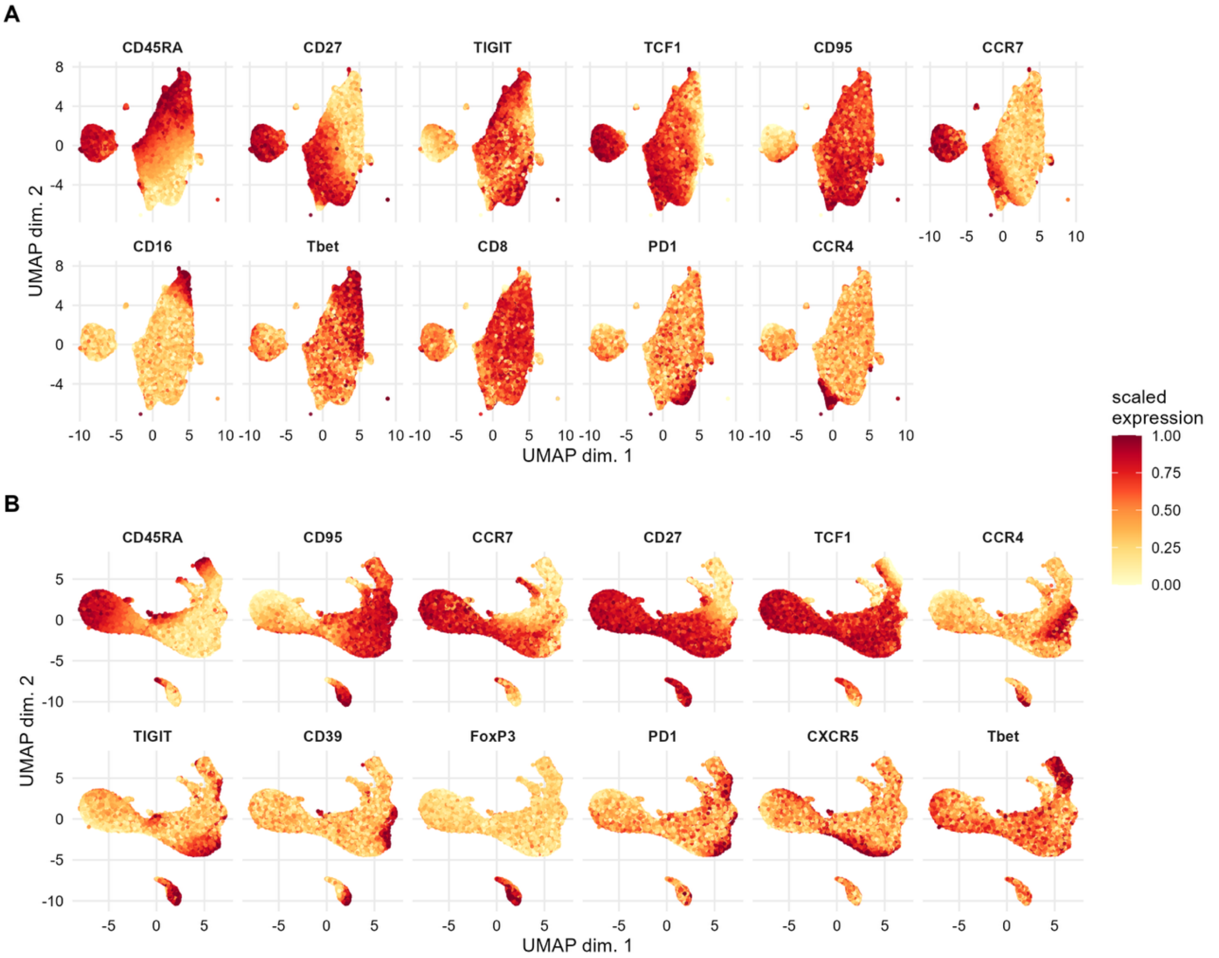

Supplementary Fig. 10. Identification of CD4 and CD8 T-cells in spectral flow cytometry ICS. UMAPs showing expression of markers used for clustering of CD8 (A) and CD4 (B) T-cells.

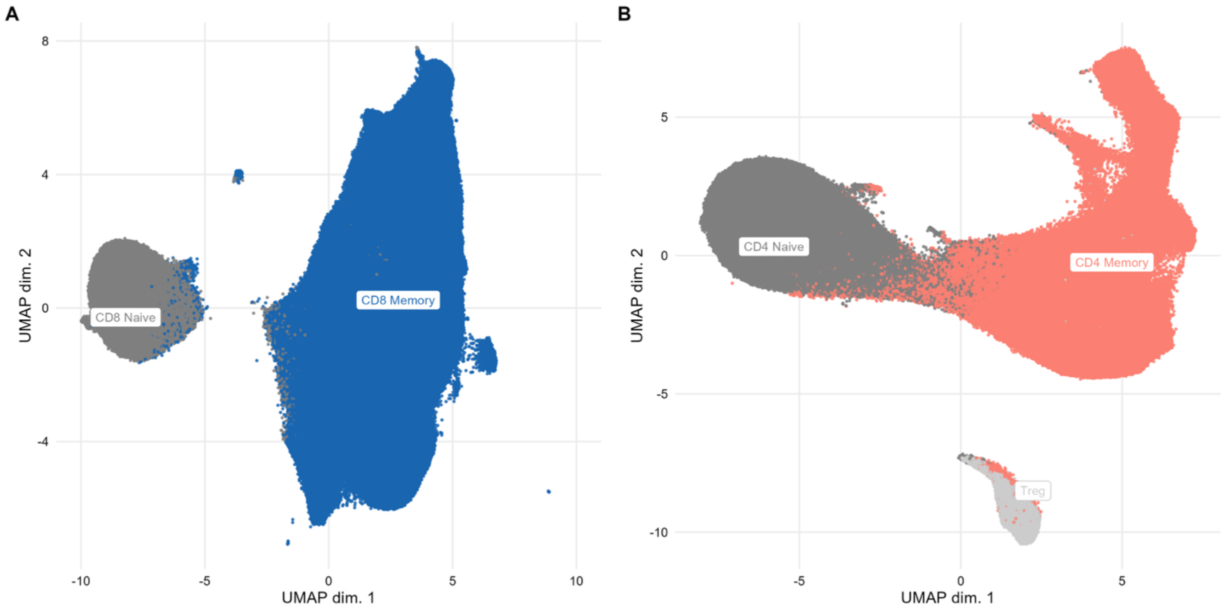

Supplementary Fig. 11. Identification of naïve and memory CD4 and CD8 T-cells in spectral flow cytometry ICS. UMAPs showing memory and naïve CD8 (A) and CD4 (B) T-cells and Tregs.

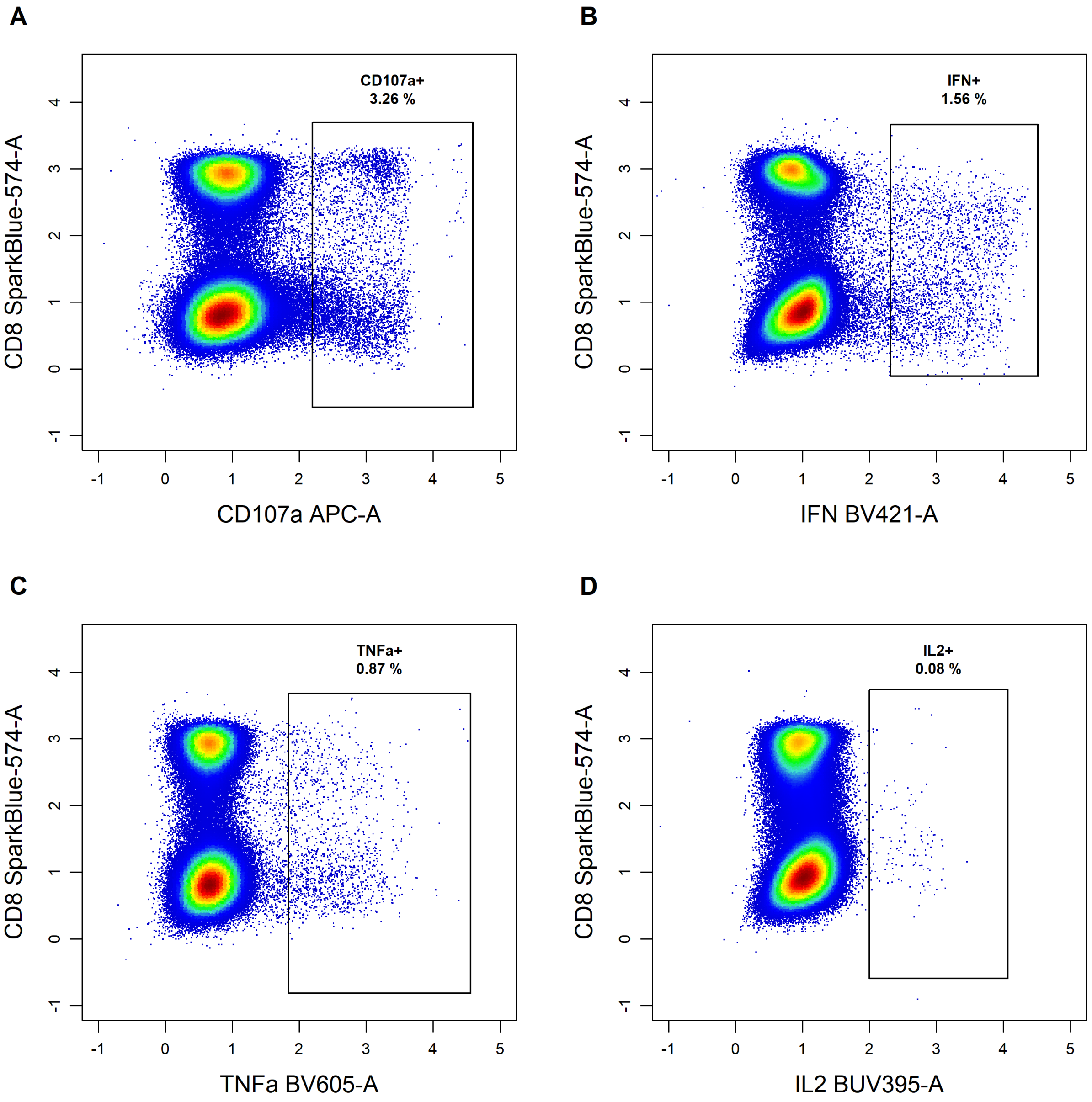

Supplementary Fig. 12. Spectral flow cytometry ICS gates for degranulation and cytokines. Representative sample showing gating of the degranulation marker CD107a (A), and the cytokines IFN-γ (B), TNF-α (C), and IL-2 (D).

References for Supplementary Information:

1. Bruner, K.M. *et al.* A quantitative approach for measuring the reservoir of latent HIV-1 proviruses. *Nature* **566**, 120-125 (2019).

2. Wei, X. *et al.* Emergence of resistant human immunodeficiency virus type 1 in patients receiving fusion inhibitor (T-20) monotherapy. *Antimicrob Agents Chemother* **46**, 1896-1905 (2002).

3. Rosás-Umbert, M. *et al.* Administration of broadly neutralizing anti-HIV-1 antibodies at ART initiation maintains long-term CD8(+) T cell immunity. *Nat Commun* **13**, 6473 (2022).

4. Hu, Y. & Smyth, G.K. ELDA: extreme limiting dilution analysis for comparing depleted and enriched populations in stem cell and other assays. *J Immunol Methods* **347**, 70-78 (2009).

5. Fisher, K. *et al.* Plasma-Derived HIV-1 Virions Contain Considerable Levels of Defective Genomes. *Journal of Virology* **96**, e02011-02021 (2022).

6. Shan, L. *et al.* A novel PCR assay for quantification of HIV-1 RNA. *J Virol* **87**, 6521-6525 (2013).

7. Cole, B. *et al.* Extensive characterization of HIV-1 reservoirs reveals links to plasma viremia before and during analytical treatment interruption. *Cell reports* **39**, 110739 (2022).

8. McMyn, N.F. *et al.* The latent reservoir of inducible, infectious HIV-1 does not decrease despite decades of antiretroviral therapy. *J Clin Invest* **133** (2023).

9. Bertagnolli, L.N. *et al.* Autologous IgG antibodies block outgrowth of a substantial but variable fraction of viruses in the latent reservoir for HIV-1. *Proceedings of the National Academy of Sciences of the United States of America* **117**, 32066-32077 (2020).

10. Gunst, J.D. *et al.* Early intervention with 3BNC117 and romidepsin at antiretroviral treatment initiation in people with HIV-1: a phase 1b/2a, randomized trial. *Nat Med* **28**, 2424-2435 (2022).

11. Shen, L. *et al.* Dose-response curve slope sets class-specific limits on inhibitory potential of anti-HIV drugs. *Nat Med* **14**, 762-766 (2008).

12. Jilek, B.L. *et al.* A quantitative basis for antiretroviral therapy for HIV-1 infection. *Nat Med* **18**, 446-451 (2012).

13. Laskey, S.B. & Siliciano, R.F. Quantitative evaluation of the antiretroviral efficacy of dolutegravir. *JCI Insight* **1**, e90033 (2016).

14. Chou, T.C. & Talalay, P. Quantitative analysis of dose-effect relationships: the combined effects of multiple drugs or enzyme inhibitors. *Adv Enzyme Regul* **22**, 27-55 (1984).

15. Niessl, J. *et al.* Combination anti-HIV-1 antibody therapy is associated with increased virus-specific T cell immunity. *Nat Med* **26**, 222-227 (2020).

16. Ellis B, H.P., Hahne F, Le Meur N, Gopalakrishnan N, Spidlen J, Jiang M, Finak G (2024),. flowCore: Basic structures for flow cytometry data. R package version 2.14.1.

17. Van P, J.W., Gottardo R, Finak G (2018),. ggcyto: Next-generation open-source visualization software for cytometry.: <https://doi.org/10.1093/bioinformatics/bty441>.

18. Emmaneel A (2023). PeacoQC: Peak-based selection of high quality cytometry data. doi:10.18129/B9.bioc.PeacoQC, R package version 1.12.0, <https://bioconductor.org/packages/PeacoQC>.

19. Crowell H, Z.V., Chevrier S, Robinson M (2023). CATALYST: Cytometry dATa anALYSis Tools. R package version 1.26.0 ed: doi: 10.18129/B9.bioc.CATALYST, R package version 1.26.0, <https://bioconductor.org/packages/CATALYST>.

20. Van Gassen, S. *et al.* FlowSOM: Using self-organizing maps for visualization and interpretation of cytometry data. *Cytometry Part A* **87**, 636-645 (2015).

21. Cillo Anthony, R. *et al.* Improved Single-Copy Assays for Quantification of Persistent HIV-1 Viremia in Patients on Suppressive Antiretroviral Therapy. *Journal of Clinical Microbiology* **52**, 3944-3951 (2014).

22. Cillo, A.R. *et al.* Plasma viremia and cellular HIV-1 DNA persist despite autologous hematopoietic stem cell transplantation for HIV-related lymphoma. *Journal of acquired immune deficiency syndromes (1999)* **63**, 438-441 (2013).

23. Guindon, S. *et al.* New algorithms and methods to estimate maximum-likelihood phylogenies: assessing the performance of PhyML 3.0. *Syst Biol* **59**, 307-321 (2010).

24. Yu, G., Smith, D.K., Zhu, H., Guan, Y. & Lam, T.T.-Y. ggtree: an r package for visualization and annotation of phylogenetic trees with their covariates and other associated data. *Methods in Ecology and Evolution* **8**, 28-36 (2017).

25. Gunst, J.D. *et al.* Impact of a TLR9 agonist and broadly neutralizing antibodies on HIV-1 persistence: the randomized phase 2a TITAN trial. *Nat Med* **29**, 2547-2558 (2023).

26. Kinloch, N.N. *et al.* HIV-1 diversity considerations in the application of the Intact Proviral DNA Assay (IPDA). *Nature Communications* **12**, 165 (2021).

27. Simonetti, F.R. *et al.* Antigen-driven clonal selection shapes the persistence of HIV-1-infected CD4+ T cells in vivo. *J Clin Invest* **131** (2021).
