# Supplementary material for: Autologous neutralizing antibodies and polyfunctional T cells contribute to long-term HIV-1 post-intervention control
Source: Nat Immunol. 2026 Mar 3;27(4):812–26. doi: 10.1038/s41590-026-02448-z (PMC13043296; doi:10.1038/s41590-026-02448-z)
Supplement: Supplementary file 2 — Reporting Summary [file 41590_2026_2448_MOESM2_ESM.pdf]

nature portfolio

Corresponding author(s): Katie Fisher

Last updated by author(s): 26/01/2026

Reporting Summary

Nature Portfolio wishes to improve the reproducibility of the work that we publish. This form provides structure for consistency and transparency in reporting. For further information on Nature Portfolio policies, see our Editorial Policies and the Editorial Policy Checklist.

Statistics

For all statistical analyses, confirm that the following items are present in the figure legend, table legend, main text, or Methods section.

n/a	Confirmed	
☐	☒	The exact sample size (*n*) for each experimental group/condition, given as a discrete number and unit of measurement
☐	☒	A statement on whether measurements were taken from distinct samples or whether the same sample was measured repeatedly
☐	☒	The statistical test(s) used AND whether they are one- or two-sided<br>*Only common tests should be described solely by name; describe more complex techniques in the Methods section.*
☐	☒	A description of all covariates tested
☐	☒	A description of any assumptions or corrections, such as tests of normality and adjustment for multiple comparisons
☐	☒	A full description of the statistical parameters including central tendency (e.g. means) or other basic estimates (e.g. regression coefficient) AND variation (e.g. standard deviation) or associated estimates of uncertainty (e.g. confidence intervals)
☐	☒	For null hypothesis testing, the test statistic (e.g. *F*, *t*, *r*) with confidence intervals, effect sizes, degrees of freedom and *P* value noted<br>*Give P values as exact values whenever suitable.*
☐	☒	For Bayesian analysis, information on the choice of priors and Markov chain Monte Carlo settings
☒	☐	For hierarchical and complex designs, identification of the appropriate level for tests and full reporting of outcomes
☒	☐	Estimates of effect sizes (e.g. Cohen's *d*, Pearson's *r*), indicating how they were calculated

Our web collection on statistics for biologists contains articles on many of the points above.

Software and code

Policy information about availability of computer code

Data collection	QuantaSoft software (Bio-Rad, version 1.7.4), 5-laser Sony ID7000 Spectral Analyser (SONY Biotechnologies, San Jose, CA), QuantStudio 7 Pro Real-Time PCR system (Applied Biosystems, A43183), LSRII flow cytometer (BD Biosciences)
Data analysis	Custom code, utilising published R packages, was used in this manuscript to analyse spectral flow cytometry data and paired single-cell transcriptome and TCR sequencing data. These codes can be found at the following page: https://github.com/SoegaardLab/2025_Fisher_Garcia_Frattari_Naasz_et_al. For analysis of spectral flow cytometry data, the following packages were used: R v.4.4.0, R version 4.3.2 with RStudio version 2023.12.1.402, flowCore v2.20.0 (https://github.com/RGLab/flowCore), flowWorkspace v4.20.0 (https://github.com/RGLab/flowWorkspace), ggcyto v1.36.1 (https://github.com/RGLab/ggcyto), PeacoQC v1.18.0 (https://github.com/saeyslab/PeacoQC), CytoExploreR v1.1.0 (https://github.com/DillonHammill/CytoExploreR), openCyto v2.20.1 (https://github.com/RGLab/openCyto), CATALYST v1.32.1 (https://github.com/HelenaLC/CATALYST), SingleCellExperiment v1.30.1 (https://github.com/drisso/SingleCellExperiment), SummarizedExperiment v1.38.1 (https://bioconductor.org/packages/release/bioc/html/SummarizedExperiment.html), FlowSOM v2.16.0 (https://www.bioconductor.org/packages/release/bioc/html/FlowSOM.html), uwot v0.2.3 (https://github.com/jlmelville/uwot) and scater v1.35.0 (https://www.bioconductor.org/packages/release/bioc/html/scater.html). For analysis of paired single-cell transcriptome and TCR sequencing data, the following packages were used: Cell Ranger v8.0.1 (10x Genomics; https://www.10xgenomics.com/support/software/cell-ranger/latest), Scanpro 0.4.0 (https://github.com/loosolab/scanpro), scDblFinder v1.18.0 (https://github.com/plger/scDblFinder), SoupX v1.6.2 (https://github.com/constantAmateur/SoupX), Seurat v5 (https://github.com/satijalab/seurat), Harmony v1.2.3 (https://github.com/immunogenomics/harmony), MAST v1.30.0 (https://github.com/RGLab/MAST), clusterProfiler v4.12.6 (https://github.com/YuLab-SMU/clusterProfiler), enrichplot v1.24.4 (https://github.com/YuLab-SMU/enrichplot), DSB v2.0.0 (https://github.com/niaid/dsb), DescTools v0.99.56 (https://cran.r-project.org/web/packages/DescTools/index.html) and Aarhus University HPC-facility GenomeDK (https://genome.au.dk/). An in-house pipeline was used for assembly of NFL HIV sequences generated by PRLS was used (https://github.com/laulambr/virus_assembly), utilising multiqc v1.23 (https://github.com/MultiQC/MultiQC), bbmap v39.01 (https://

github.com/BioInfoTools/BBMap), samtools v1.18 (https://github.com/samtools/samtools), qualimap v2.3 (https://anaconda.org/channels/bioconda/packages/qualimap/overview), megahit v1.2.9 (https://github.com/voutcn/megahit), blast v1.16.0, mafft v7.520 (https://mafft.cbrc.jp/alignment/software/), and seqtk v1.4 (https://github.com/lh3/seqtk). An in-house pipeline for assembly and classification of HIV NFL sequences by Q4PCR (https://github.com/stratust/DIHIVA), utilising BBtools package v38.72 (https://sourceforge.net/projects/bbmap/), Trim Galore package v0.6.4 (https://github.com/FelixKrueger/TrimGalore) and SPAdes v3.13.1 (https://github.com/ablab/spades). An in-house intactness pipeline was used for classification of NFL HIV sequences generated by MIP-seq (https://github.com/BWH-Lichterfeld-Lab/Intactness-Pipeline). Additional code used in the paper has been cited appropriately. Additional tools and packages used for analysis: bwa-mem v0.7.19 (https://github.com/lh3/bwa), Ensembl (v113, www.ensembl.org), the UCSC Genome Browser (www.genome.ucsc.edu), GENCODE (v47, www.gencodegenes.org), Los Alamos webtools ElimDupes (https://www.hiv.lanl.gov/content/sequence/elimdupesv2/elimdupes.html), GeneCutter (https://www.hiv.lanl.gov/content/sequence/GENE_CUTTER/cutter.html), Hypermut v3.0 (https://www.hiv.lanl.gov/content/sequence/HYPERMUT/hypermutv3.html) and Highlighter (https://www.hiv.lanl.gov/content/sequence/HIGHLIGHT/highlighter_top.html), and the HIV Molecular Immunology Database Search (https://www.hiv.lanl.gov/mojo/immunology/search/ctl/form.html), PhyML v3.3.20220408 (https://github.com/stephaneguindon/phyml), ggTree v3.12.0 (https://guangchuangyu.github.io/software/ggtree/), Geneious Prime v2025.1.2, FlowJo v10.10.0 and v10.5.0 Software (BD Biosciences), ID7000 Software version 2.0.2 (SONY Biotechnologies, San Jose, CA), GraphPad Prism v 10.2.0, IUPM algorithm (http://silicianolab.johnshopkins.edu), bNAb-ReP v1.1-4 (https://github.com/RedaRawi/bNAb-ReP), the Los Alamos "Best-defined CTL/CD8+ Epitope Summary" (https://www.hiv.lanl.gov/content/immunology/tables/optimal_ctl_summary.html), Los Alamos "CTL/CD8+ Epitope Variants and Escape Mutations" table (http://www.hiv.lanl.gov/content/immunology/variants/ctl_variant.html) and the Biopython SeqUtils package (https://github.com/biopython/biopython/tree/master/Bio/SeqUtils).

For manuscripts utilizing custom algorithms or software that are central to the research but not yet described in published literature, software must be made available to editors and reviewers. We strongly encourage code deposition in a community repository (e.g. GitHub). See the Nature Portfolio guidelines for submitting code & software for further information.

Data

Policy information about availability of data

All manuscripts must include a data availability statement. This statement should provide the following information, where applicable:

- Accession codes, unique identifiers, or web links for publicly available datasets
- A description of any restrictions on data availability
- For clinical datasets or third party data, please ensure that the statement adheres to our policy

HIV-1 proviral, qVOA and plasma-derived sequences have been deposited in GenBank with the accession numbers PX465444-PX466083, MK115946-MK116091, MN090734-MN090850 and MW063053-MW063065. HIV-1 env sequences utilised to check for superinfection can be found in Genbank with the accession numbers OR014534-OR014555, OR014635-OR014658, OR014662-OR014681, OR014984-OR015005, OR015084-OR015110, OR015113-OR015155 and PX892135-PX892225. Paired single-cell transcriptome and TCR sequencing data has been uploaded to the European Genome-Phenome Archive (accession number EGAS50000001570). Access to data will be assessed on a case-by-case basis and available upon signing of a Data Access Agreement. Any additional information required can be accessed by contacting the corresponding author, Ole Schmeltz Søgaard (olesoega@rm.dk).

Research involving human participants, their data, or biological material

Policy information about studies with human participants or human data. See also policy information about sex, gender (identity/presentation), and sexual orientation and race, ethnicity and racism.

Reporting on sex and gender	People living with HIV-1 included in this manuscript included three post-intervention controllers and six ART-suppressed individuals/non-controllers, 5/6 of which were of male sex (self-reported/identified from medical records). One additional progressor individual with HIV-1 was also included in the manuscript, who was of female sex. Due to the nature of the study investigating post-intervention control of HIV-1, investigations could only utilise sampling from identified post-intervention controllers for whom samples were available, all of which were male. Secondary use of samples from ART-suppressed individuals who participated in the eCLEAR trial was restricted by availability of biological sampling.
Reporting on race, ethnicity, or other socially relevant groupings	Due to the nature of our investigation into post-intervention control of HIV-1, only a small number of post-intervention controllers (and an appropriate number of non-controlling individuals) were included in the study. This is because investigations could only utilise sampling from identified post-intervention controllers for whom samples were available. Information regarding the race/ethnicity of all individuals included in the Methods section and Supplementary Tables 1 and 5 of the manuscript.
Population characteristics	Our manuscript is based on the identification of three cases of HIV-1 post-intervention control. Therefore, no specific measures could be taken to ensure equal distribution of population characteristics as investigation was based on sample availability. Population characteristics of all individuals can be found in Supplementary Tables 1 and 5.
Recruitment	Our manuscript is based on the identification of three cases of HIV-1 post-intervention control. Therefore, specific recruitment into this study is not relevant.
Ethics oversight	National Committee on Health Research Ethics in Denmark (continued ATI, leukapheresis and secondary use of samples from eCLEAR clinical trial), Ethics Committee of the Medical Faculty of the University of Cologne, the George Washington University Institution Review Board and Weill Cornell Medicine Institutional Review Board. Written informed consent was given by all individuals.

Note that full information on the approval of the study protocol must also be provided in the manuscript.

Field-specific reporting

Please select the one below that is the best fit for your research. If you are not sure, read the appropriate sections before making your selection.

☒ Life sciences ☐ Behavioural & social sciences ☐ Ecological, evolutionary & environmental sciences

For a reference copy of the document with all sections, see nature.com/documents/nr-reporting-summary-flat.pdf

Life sciences study design

All studies must disclose on these points even when the disclosure is negative.

Sample size	Sample size calculations are not appropriate for our study as the design was based on three identified HIV-1 post-intervention controllers.
Data exclusions	Data were only excluded on the basis of insufficient material available to run assays at certain timepoints.
Replication	All assays were performed once at each timepoint due to limited biological sample availability, except for neutralisation experiments which were performed using duplicates or triplicates. However, multiple assays were used at multiple timepoints to corroborate our findings and claims. For the participant-derived xenograft experiments, a total of 19 mice were included in the study.
Randomization	For all experiments involving sampling from people with HIV-1, randomisation was not appropriate as the study was based on three identified HIV-1 post-intervention controllers, and included six ART-suppressed/non-controller individuals for comparison. For the participant-derived xenograft experiments, mice were divided into two groups (one to receive autologous memory CD8 T cells from ID107 and one who did not receive memory CD8 T cells) following infection of all mice with autologous virus. The division of the two groups ensured equal distribution of plasma HIV-1 viral loads and CD4 T cell counts following establishment of infection, and therefore was not randomised.
Blinding	All participants were assigned unique IDs prior to participation in the previous clinical trials, and these were utilised while conducting the experiments included in this manuscript. These IDs were used for identification of all samples while experiments were conducted, and therefore blinding was not relevant to this manuscript.

Reporting for specific materials, systems and methods

We require information from authors about some types of materials, experimental systems and methods used in many studies. Here, indicate whether each material, system or method listed is relevant to your study. If you are not sure if a list item applies to your research, read the appropriate section before selecting a response.

Materials & experimental systems

n/a	Involved in the study	
☐	☒	Antibodies
☐	☒	Eukaryotic cell lines
☒	☐	Palaeontology and archaeology
☐	☒	Animals and other organisms
☐	☒	Clinical data
☒	☐	Dual use research of concern
☒	☐	Plants

Methods

n/a	Involved in the study	
☒	☐	ChIP-seq
☐	☒	Flow cytometry
☒	☐	MRI-based neuroimaging

Antibodies

Antibodies used

1. CD3: PE/Dazzle 594, OKT3, Biolegend, catalog #317345, lot #B443448
2. CD4: PE/Fire 700, SK3, Biolegend, catalog #344665, lot #B437043
3. CD8: APC, SK1, Biolegend, catalog #344721, lot #B441271
4. CD3: PerCP/Cy5.5, SK7, BioLegend, catalog #300429, lot #B354280
5. CD4: BV650, RPA-T4, BioLegend, catalog #300535, lot #B366745
6. CD8: BV605, RPA-T8, BioLegend, catalog #301039, lot #B435856
7. 4-1BB: PE, 4B4-1, BioLegend, catalog #309803, lot #B367290
8. CD69: APC, FN50, BioLegend, catalog #310909, lot #B359249
9. PD-L1: BV421, B7-H1, BioLegend, catalog #374507, lot #B388684
10. IL2: BUV395, MQ1-17H12, eBioscience, catalog #363-7029-42, lot #2851058
11. CD4: BUV496, SK3, BD bioscience, catalog #612936, lot #3247822, 4204806
12. CD16: BUV563, 3G8, BD bioscience, catalog #568289, lot #4095102, 4124989, 4185605
13. CD3: BUV615, SK7, BD bioscience, catalog #751252, lot #4243045, 4243046, 4243047
14. CXCR5: BUV661, RF8B2, BD bioscience, catalog #741559, lot #5058675
15. Tbet: BUV737, O4-46, BD bioscience, catalog #568166, lot #4102178, 4178340
16. IFNg: BV421, B27, BD bioscience, catalog #562988, lot #4234096
17. CCR4: BV510, L291H4, Biolegend, catalog #359416, lot #B377907
18. CD19, BV570, HIB19, Biolegend, catalog #302236, lot #B386458

19. TNFa, BV605, Mab11, Biolegend, catalog #502936, lot #B422747
20. CD27: BV650, O323, Biolegend, catalog #302828, lot #B375145, B412778
21. PD-1: BV711, EH12.2H7, Biolegend, catalog #329928, lot #B410254
22. CCR7: BV785, G043H7, Biolegend, catalog #353230, lot #B415814
23. Granulysin: AF488, RB1, BD bioscience, catalog #558254, lot #333150
24. CD8: Sparkblue-574, SK1, Biolegend, catalog #344786, lot #B430783
25. Granzyme B: PerCP, QA18A28, Biolegend, catalog #396416, lot #B429745
26. TCF1: Realblue705, S33-966, BD bioscience, catalog #570635, lot #418397
27. CD45RA: Realblue780, HI100, BD bioscience, catalog #569081, lot #3341167, 4151115
28. IL4: PE, MP4-25D2, Biolegend, catalog #500810, lot #255580
29. IL13: PE, JES10-5A2, Biolegend, catalog #501903, lot #3109515, B380784
30. Ki67: Pe-dazzle594, ki67, Biolegend, catalog #35053431, lot #B376072
31. CD95: PE-fire640, DX2, Biolegend, catalog #305658, lot #B420907
32. Granzyme K: PE-Cy7, GM26E7, Biolegend. Catalog #370516, lot #B424505
33. CD39: Pe-fire810, A1, Biolegend, catalog #328245, lot #B427223
34. CD107a: APC, H4A3, Miltenyi, catalog #130-119-869, lot #5240808332
35. FoxP3: SparkNIR-685, 206D, Biolegend, catalog #320130, lot #B42255
36. TIGIT: R718, TgMab-2, BD bioscience, catalog #569038, lot #4304734
37. Perforin: APC-fire750, B-D48, Biolegend #353318, lot #B400537
38. CD3: PerCP/eFluor710, SK7, eBioscience #46-0036, lot #1941534
39. CD4: BUV496, SK3, BD Biosciences #564651, lot #9080989
40. CD8: BV711, RPA-T8, BioLegend #301044, lot #B237121
41. 4-1BB: PE-Cy7, 4B4-1, BioLegend #309818, lot #B258325
42. CD69: BUV395, FN50, BD Biosciences #564364, lot #564364
43. PD-L1: BV421, 29E2A3, BioLegend #329714, lot #B258010

Validation

All verifications below are as found on the relevant manufacturers' website.
1. CD3 PE/Dazzle 594; Verified reativity: Human (Biolegend); Application: Flow cytometry (Quality tested, Biolegend)
2. CD4 PE/Fire 700; Verified reativity: Human (Biolegend); Application: Flow cytometry (Quality tested, Biolegend)
3. CD8 APC, SK1; Verified reativity: Human, Cynomolgus, Rhesus (Biolegend); Application: Flow cytometry (Quality tested, Biolegend)
4. CD3 PerCP/Cy5.5, SK7; Verified reativity: Human (Biolegend); Application: Flow cytometry (Quality tested, Biolegend)
5. CD4 BV650, RPA-T4; Verified reativity: Human (Biolegend); Application: Flow cytometry (Quality tested, Biolegend)
6. CD8 BV605, RPA-T8; Verified reativity: Human, Cynomolgus, Rhesus (Biolegend); Application: Flow cytometry (Quality tested, Biolegend)
7. 4-1BB PE, 4B4-1; Verified reativity: Human (Biolegend); Application: Flow cytometry (Quality tested, Biolegend)
8. CD69 APC, FN50; Verified reativity: Human (Biolegend); Application: Flow cytometry (Quality tested, Biolegend)
9. PD-L1 BV421, B7-H1; Verified reativity: Human (Biolegend); Application: Flow cytometry (Quality tested, Biolegend)
10. IL2: BUV395. Human. Applications Tested: Tested by intracellular staining followed by flow cytometry
11. CD4: BUV496. Human (QC Testing), Flow cytometry (Routinely Tested)
12. CD16: BUV563. Human (QC Testing), Rhesus, Cynomolgus, Baboon (Tested in Development). Flow cytometry (Routinely Tested)
13. CD3: BUV615. Human (Tested in Development). Flow cytometry (Qualified)
14. CXCR5: BUV661. Human (Tested in Development). Flow cytometry (Qualified)
15. Tbet: BUV737. Human (QC Testing), Mouse (Tested in Development). Intracellular staining (flow cytometry) (Routinely Tested)
16. IFNg: BV421. Human (QC Testing), Rhesus, Cynomolgus, Baboon (Tested in Development). Intracellular staining (flow cytometry) (Routinely Tested)
17. CCR4: BV510. Verified Reactivity Human. Flow cytometry (Qualified)
18. CD19, BV570. Verified Reactivity Human. Flow cytometry (Qualified)
19. TNFa, BV605. Verified Reactivity Human. Intracellular Flow cytometry (Qualified)
20. CD27: BV650. Verified Reactivity Human, Cynomolgus, Rhesus. Flow cytometry (Qualified)
21. PD-1: BV711. Verified Reactivity Human. Flow cytometry (Qualified).
22. CCR7: BV785. Verified Reactivity Human. Flow cytometry (Qualified).
23. Granulysin: AF488. Human (QC Testing). Intracellular staining (flow cytometry) (Routinely Tested)
24. CD8: Sparkblue-574. Verified Reactivity Human, Cynomolgus, Rhesus. Flow cytometry (Qualified).
25. Granzyme B: PerCP. Verified Reactivity Human, mouse. Intracellular Flow cytometry (Qualified)
26. TCF1: Realblue705. Mouse (QC Testing), Human (Tested in Development). Intracellular staining (flow cytometry) (Routinely Tested)
27. CD45RA: Realblue780. Human (QC Testing), Flow cytometry (Routinely Tested)
28. IL4: PE. Verified Reactivity Human. Intracellular Flow cytometry (Qualified)
29. IL13: PE. Verified Reactivity Human. Intracellular Flow cytometry (Qualified)
30. Ki67: Pe-dazzle594. Verified Reactivity Human. Intracellular Flow cytometry (Qualified)
31. CD95: PE-fire640. Verified Reactivity Human, Cynomolgus, Rhesus. Flow cytometry (Qualified).
32. Granzyme K: PE-Cy7. Verified Reactivity Human. Intracellular Flow cytometry (Qualified)
33. CD39: Pe-fire810. Verified Reactivity Human, Cynomolgus, Rhesus. Flow cytometry (Qualified).
34. CD107a: APC. Species tested: Human. Compatible applications. IF, IHC, ICC.
35. FoxP3: SparkNIR-685. Verified Reactivity Human. Intracellular Flow cytometry (Qualified)
36. TIGIT: R718. Human (QC Testing), Flow cytometry (Routinely Tested)
37. Perforin: APC-fire750. Verified Reactivity Human. Intracellular Flow cytometry (Qualified)
38. CD3: Verified reactivity: Human, Chimpanzee. Application: Flow Cytometry.
39. CD4: Verified reactivity: human. Application: Flow cytometry (routinely tested)
40. CD8: Verified reactivity: Human, Cynomolgus, Rhesus. Application: Flow Cytometry (Quality tested)
41. 4-1BB: Verified reactivity: Human. Application: Flow Cytometry (Quality Tested)
42: CD69: Reactivity: Human (QC Testing), Rhesus, Cynomolgus, Baboon (Tested in Development). Application: Flow Cytometry (Routinely Tested)
43: PD-L1: Verified reactivity: Human. Application: Flow Cytometry (Quality Tested)

Eukaryotic cell lines

Policy information about cell lines and Sex and Gender in Research

Cell line source(s)	TZM-bl cells (Cat No. 8129) and MOLT-4/CCR5 cell lines (Cat No 4984) were both obtained from the NIH HIV Reagent Program (https://www.beiresources.org/HIV.aspx).
Authentication	The cell lines above were not authenticated.
Mycoplasma contamination	TZM-bl cells and MOLT-4/CCR5 cell lines are negative for mycoplasma.
Commonly misidentified lines (See ICLAC register)	No commonly misidentified cell lines were used in this study.

Animals and other research organisms

Policy information about studies involving animals; ARRIVE guidelines recommended for reporting animal research, and Sex and Gender in Research

Laboratory animals	NOD.Cg-Prkdcscid Il2rgtm1Wjl/SzJ mice (stock 005557), commonly referred to as NSG mice, were purchased from The Jackson Laboratory. Female (6–8-week-old) NSG mice were used in all studies. Mice were co-housed in ventilated cages with wood chip bedding and maintained in a temperature-controlled environment with a 12-h light/dark cycle at the facilities of Weill Cornell Medical College. The temperature of the holding rooms are maintained between 70-74 degrees Fahrenheit. The relative humidity is maintained between 30% – 70%.
Wild animals	The study did not involve wild animals.
Reporting on sex	Only female mice were included in this study as all mice need to be the same sex to ensure even distribution of variables such as plasma HIV viral load and CD4 T cell count when assigning groups for the engraftment of memory CD8 T cells. Additionally, all mice are of the same sex to ensure no impact of the sex of the mouse on the assessment of function of human immune cells within the experimental conditions.
Field-collected samples	The study did not involve sample collection from the field.
Ethics oversight	All animal procedures were conducted according to a protocol approved by the Weill Cornell Medical College Institutional Animal Care and Use Committee (protocol 2018-0027).

Note that full information on the approval of the study protocol must also be provided in the manuscript.

Clinical data

Policy information about clinical studies

All manuscripts should comply with the ICMJE guidelines for publication of clinical research and a completed CONSORT checklist must be included with all submissions.

Clinical trial registration	This manuscript does not report on the findings of any clinical trials. However, the people with HIV-1 who were included in the manuscript were originally enrolled in other clinical trials.<br>eCLEAR (IDs 107, 104, 112, 110, 702, 120 and 209): EudraCT:2015-002234-53, Clinicaltrials.gov: NCT03041012<br>TITAN (ID142): EudraCT: 2018-001165-16, Clinicaltrials.gov: NCT03837756<br>ID9254: EudraCT: 2016-002803-25, Clinicaltrials.gov: NCT02825797
Study protocol	Study protocols are available upon request.
Data collection	Participants ID107, ID104, ID112, ID110, ID702, ID120 and ID209 were enrolled in the eCLEAR trial. ID107 continued to be followed longitudinally following completion of the eCLEAR trial until 2025. ID142 was originally enrolled in the TITAN trial, and continued to be followed longitudinally until 2024. Continued ATI for ID107 and ID142 was done under the trial protocol and leukapheresis was done according to the National Committee on Health Research Ethics in Denmark (#1-10-72-38-23). Secondary use of samples from individuals who participated in eCLEAR was approved by the National Committee on Health Research Ethics in Denmark (1-10-72-110-16). ID9254 was enrolled in a different clinical trial, and continued to be monitored longitudinally until 2025. Research blood samples were collected under an observational protocol approved by the Ethics Committee of the Medical Faculty of the University of Cologne (UKK #16-054).
Outcomes	Outcomes of clinical trials are not relevant for this manuscript.

Plants

Seed stocks

Report on the source of all seed stocks or other plant material used. If applicable, state the seed stock centre and catalogue number. If plant specimens were collected from the field, describe the collection location, date and sampling procedures.

Novel plant genotypes

Describe the methods by which all novel plant genotypes were produced. This includes those generated by transgenic approaches, gene editing, chemical/radiation-based mutagenesis and hybridization. For transgenic lines, describe the transformation method, the number of independent lines analyzed and the generation upon which experiments were performed. For gene-edited lines, describe the editor used, the endogenous sequence targeted for editing, the targeting guide RNA sequence (if applicable) and how the editor was applied.

Authentication

Describe any authentication procedures for each seed stock used or novel genotype generated. Describe any experiments used to assess the effect of a mutation and, where applicable, how potential secondary effects (e.g. second site T-DNA insertions, mosiacism, off-target gene editing) were examined.

Flow Cytometry

Plots

Confirm that:

☒ The axis labels state the marker and fluorochrome used (e.g. CD4-FITC).

☒ The axis scales are clearly visible. Include numbers along axes only for bottom left plot of group (a 'group' is an analysis of identical markers).

☒ All plots are contour plots with outliers or pseudocolor plots.

☒ A numerical value for number of cells or percentage (with statistics) is provided.

Methodology

Sample preparation

Cryopreserved PBMCs were thawed and washed in RPMI supplemented with FBS and P/S. Treatment of samples following thawing and washing depended on the experiment performed (i.e. lymphocyte proliferation assay, AIM assay or spectral flow cytometry intracellular cytokine staining assay), and is described in the Methods section.

Instrument

MACSQuant16 (Miltenyi Biotec) (lymphocyte proliferation assay, AIM assay), MACSQuant Tyto Cell Sorter (Miltenyi Biotec) (sorting of AIM+ cells), 5-laser Sony ID7000 Spectral Analyser (SONY Biotechnologies, San Jose, CA) (spectral flow cytometry), LSRII flow cytometer (AIM assay).

Software

Flow data acquired for the AIM and lymphocyte proliferation assay was analysed using FlowJo 10.10.0 or FlowJo 10.5.0. Spectral flow unmixing was performed using ID7000 Software version 2.0.2 (SONY Biotechnologies, San Jose, CA). All analyses downstream of spectral unmixing were conducted in R version 4.3.2 with RStudio version 2023.12.1.402 using an in-house pipeline built through the assembly of several algorithms (described in the Methods section).

Cell population abundance

200,000 PBMCs were seeded in each well for stimulation in the lymphocyte proliferation assay. 1 x 10^6 PBMCs were seeded in each well for the AIM assay for ID107 and ID142, and at 10 x 10^6 cells/mL in each well for the AIM assay for ID9254. For spectral flow cytometry ICS, the input ranged from 0.8 x 10^6 PBMCs to 1.3 x 10^6 PBMCs.

Gating strategy

In the lymphocyte proliferation assay, cells were gated as follows: Live cells were gated as the negative population in a dead cell stain. Single cells were gated in a SSC-A/SSC-H plot. Lymphocytes were gated in a SSC-A/FSC-A plot. CD3 postive cells were gated in a SSC-A/CD3-A. Single positive CD4 and CD8 cells were gated in a CD8-A/CD4-A. Percentage proliferating cells was gated for both CD4 and CD8 single positive cells as populations dimmer than the undivided peak.
In the AIM assay for ID107 and ID142, cells were gated as follows: Live cells were gated as the negative population in a dead cell stain. Single cells were gated in a SSC-A/SSC-H plot. Lymphocytes were gated in a SSC-A/FSC-A plot. CD3 postive cells were gated in a SSC-A/CD3-A. Single positive CD4 and CD8 cells were gated in a CD8-A/CD4-A. Boolean gating of the three AIMs was then applied to both CD4 and CD8 positive cells to detect double- and triple positive cells. For the AIM assay for ID9254, the gating strategy is reported in Niessl et al., Nature Medicine, 2020.
For spectral flow cytometry, a detailed description of the gating strategy is given in the Methods section. In brief, cells were pregated in a SSC-A/FSC-A plot to identify cells and remove debris, live cells were gated as the negative population in a dead cell stain, singlets in a FSC-A/FSC-H plot, and lymphocytes in a FSC-A/SSC-A plot. The lymphocyte population was then clustered into main populations using canonical lineage markers (CD3, CD8, CD4, CD16, CD19). Memory CD4 and CD8 T-cell clusters were identified by their expression of CD45RA, CCR7, CD27, and CD95, and cells were gated for the degranulation marker CD107a and the cytokines IFN-g, TNF-a and IL-2.

☒ Tick this box to confirm that a figure exemplifying the gating strategy is provided in the Supplementary Information.
